# Supplementary material for: Combining pangenomics and population genetics finds chromosomal re-arrangements, diversified chromosome segments, copy number variations and transposon polymorphisms in wheat and rye powdery mildew
Source: PLoS Pathog. 2026 Apr 20;22(4):e1013196. doi: 10.1371/journal.ppat.1013196 (PMC13124064; doi:10.1371/journal.ppat.1013196)
Supplement: S1 Appendix — Fig A. PCA variance. Percentage of variance explained for the PCA in Fig 1. Fig B. Cross validation error for the ADMIXTURE analysis (for number of ancestries/“K” from 2 to 20). Fig C. Admixture plot of the 399 isolates for K = 1 through K = 12. Fig D. Singleton analysis of the B. graminis populations. Singletons of 400 isolates or a subset, excluding most of the clonal isolates and a few ones with very low coverage. (A) Singletons of both SNPs and INDELs for a subset of the eight isolates that were used as random ones to make up populations. Their names are listed. (B) Singleton SNPs of all 400 isolates. (C) Singleton INDELs for all 400 isolates. Fig E. Mantel test on subsets of B. graminis isolates. Mantel test using the genetic data from the SNPs and the coordinates for the geographic correlation for a worldwide dataset where geographic information is available (on the left) and for the area around the region of origin (on the right). The colors represent two-dimensional kernel density estimation of the points with red (higher density), yellow (average density), blue (lower density) and white (no density). Fig F. Distribution of the LINE RII_Fuji in the genomes of three powdery mildews. Distribution in: (A) The B.g. secalis Bgs_1451 genome, (B) the B.g. triticale Bgtl_THUN_12 genome, and (C) the B.g. tritici Bgt_CHE_96224 genome. Fig G. BUSCO results of the pangenome. BUSCO analyses results for all the wheat and rye powdery mildew genomes that have been long read sequenced with PacBio and assembled into chromosomes on three different levels of databases being used: (A) Fungi, (B) Ascomycota, and (C) Leotiomycetes. Fig H. BUSCO results of the pangenome’s CDS. BUSCO analyses results for all the wheat and rye powdery mildew coding sequences (CDS) that have been analysed with maker using the near-chromosome scale assemblies, on three different levels of databases being used: (A) Fungi, (B) Ascomycota, and (C) Leotiomycetes. Fig I. Gene annotation statistics. (A) Numb [file ppat.1013196.s001.pdf]

Supplementary Material

S1 Appendix

Supplementary Notes

Note A.

In the greater chromosomal region (at about 525 kb) at the left side of chr-11 that has been rearranged with an origin from *B.g. secalis* in *B.g. triticale* (Fig 4A), there was a candidate effector, BgTH12-07698 which had no exact protein homologs in Bgt_CHE_96224 (homologs: defined as having amino acid identity higher than 70%), and which has an identical DNA sequence with the rye powdery mildew gene *Bgs1459-09656*. This candidate gene showed expression in BgtI_THUN_12 (Figure U.A in S1 Appendix). The gene could not be found in any of the wheat powdery mildew genomes. However, there was some protein homology (~67% amino acid identity) with a protein from the effector family E003 in *B.g. tritici* isolates (e.g. BgtE-20034 and BgUSA2-05770), and less (>50%) with the rest of the proteins in the same family (Figure U.B-U.C in S1 Appendix). This protein with ~64 to ~67% identity was found in chromosome 7 in the isolates and the Bgs_1459 and BgtI_THUN_12 isolates have again identical such proteins (BgTH12-02784 and Bgs1459-05507) and between these and the *B.g. tritici* isolates there were between 2 and 6 amino acid changes.

Supplementary Figures

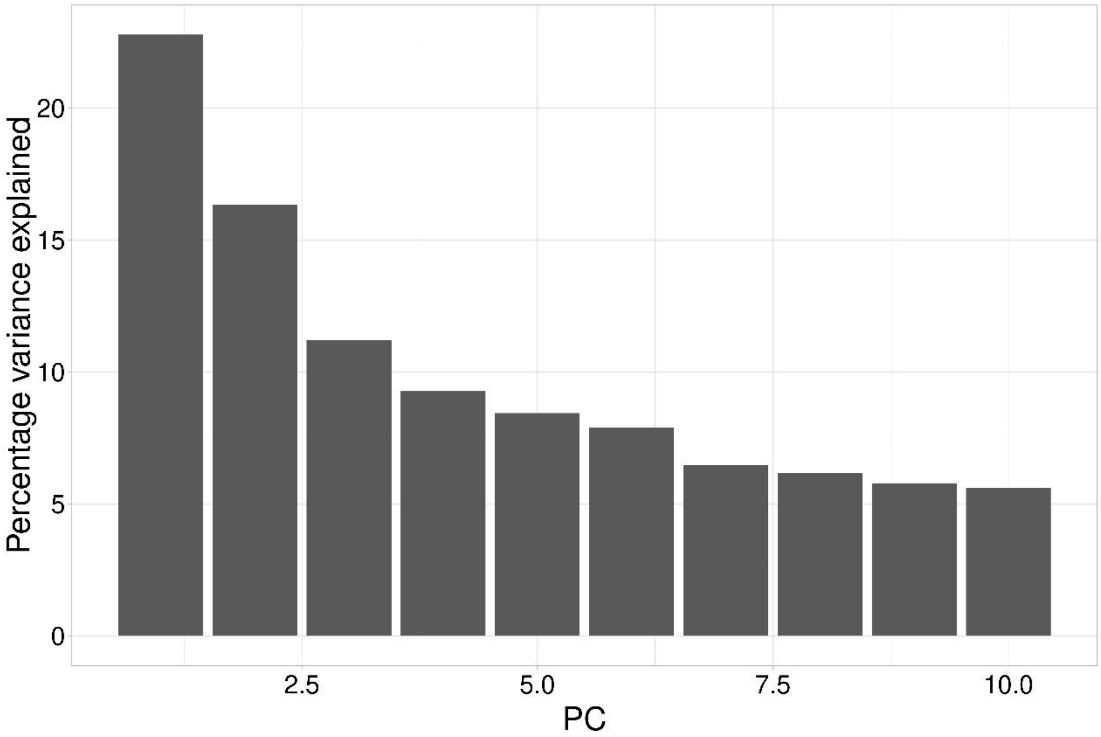

Figure A. PCA variance.

Percentage of variance explained for the PCA in Fig 1.

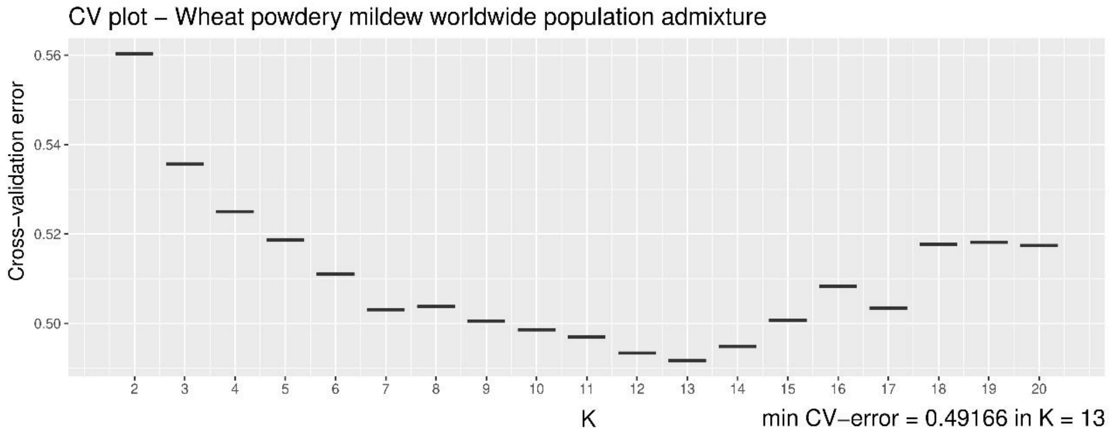

Figure B. Cross validation error for the ADMIXTURE analysis (for number of ancestries/“K” from 2 to 20).

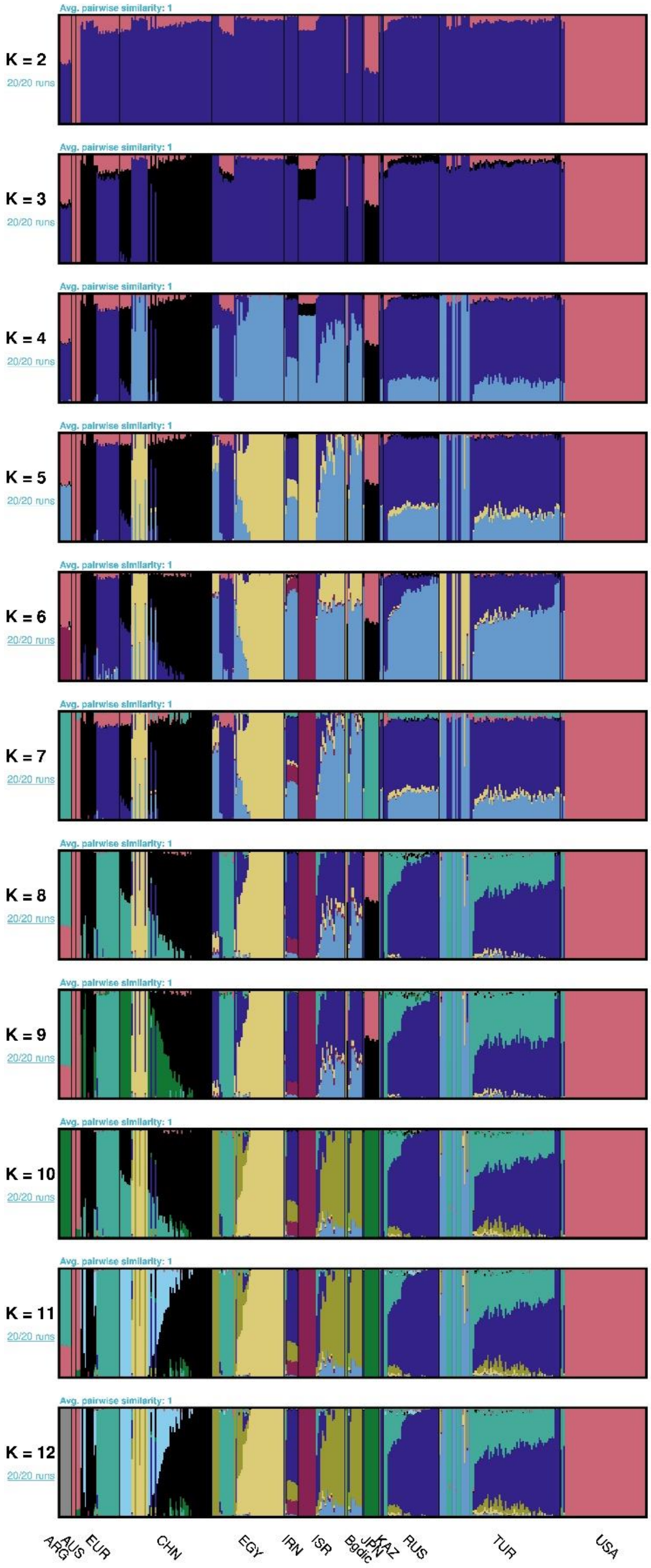

Figure C. Admixture plot of the 399 isolates for K=1 through K=12.

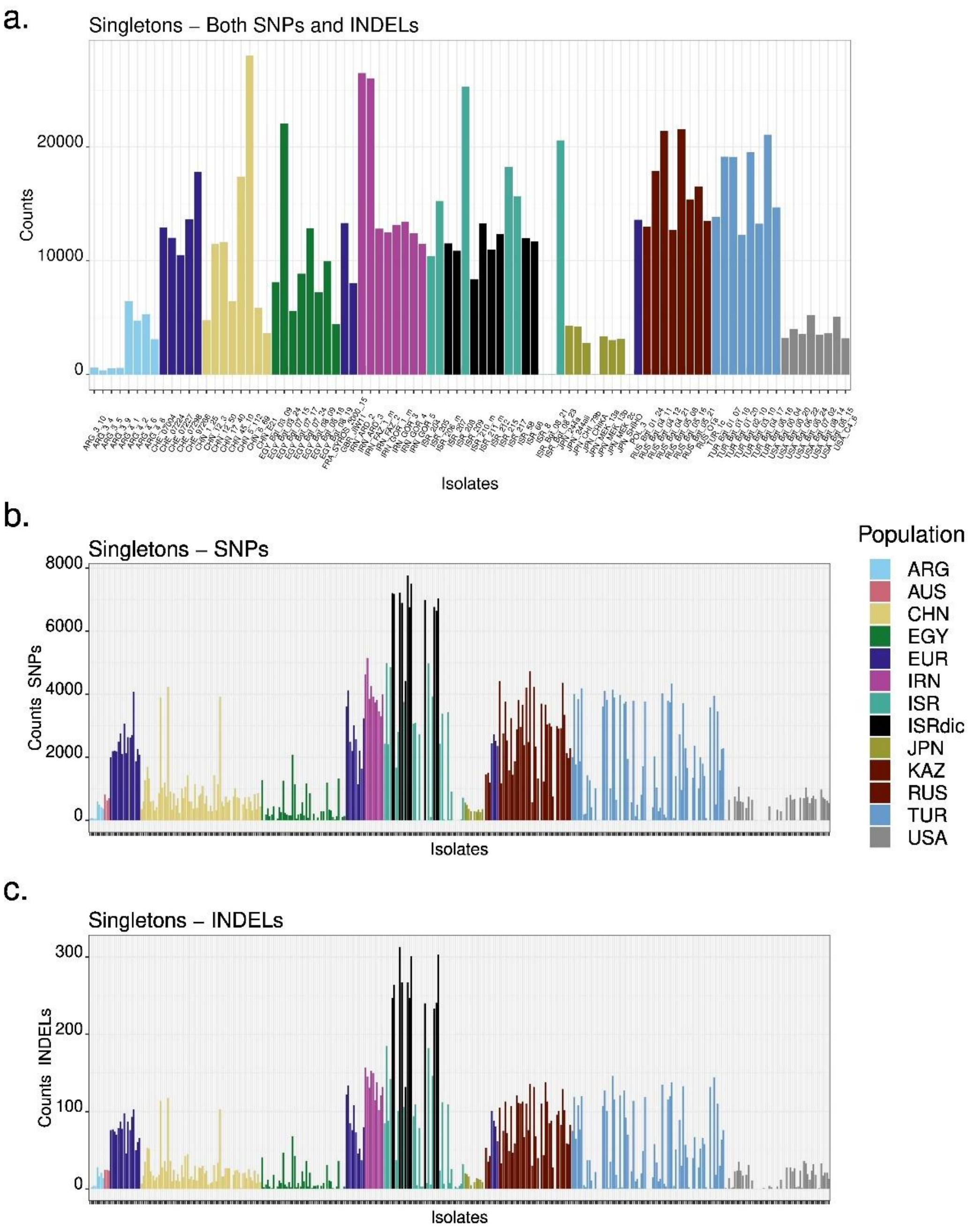

Figure D. Singleton analysis of the *B. graminis* populations.

Singletons of 400 isolates or a subset, excluding most of the clonal isolates and a few ones with very low coverage. (A) Singletons of both SNPs and INDELs for a subset of the eight isolates that were used as random ones to make up populations. Their names are listed. (B) Singleton SNPs of all 400 isolates. (C) Singleton INDELs for all 400 isolates.

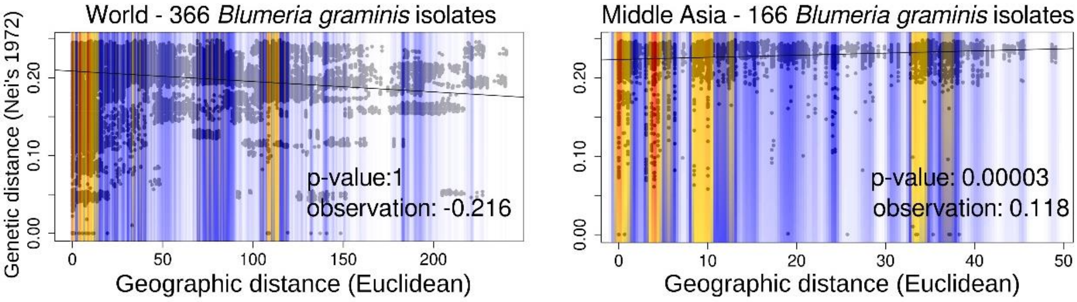

Figure E. Mantel test on subsets of *B. graminis* isolates.

Mantel test using the genetic data from the SNPs and the coordinates for the geographic correlation for a worldwide dataset where geographic information is available (on the left) and for the area around the region of origin (on the right). The colors represent two-dimensional kernel density estimation of the points with red (higher density), yellow (average density), blue (lower density) and white (no density).

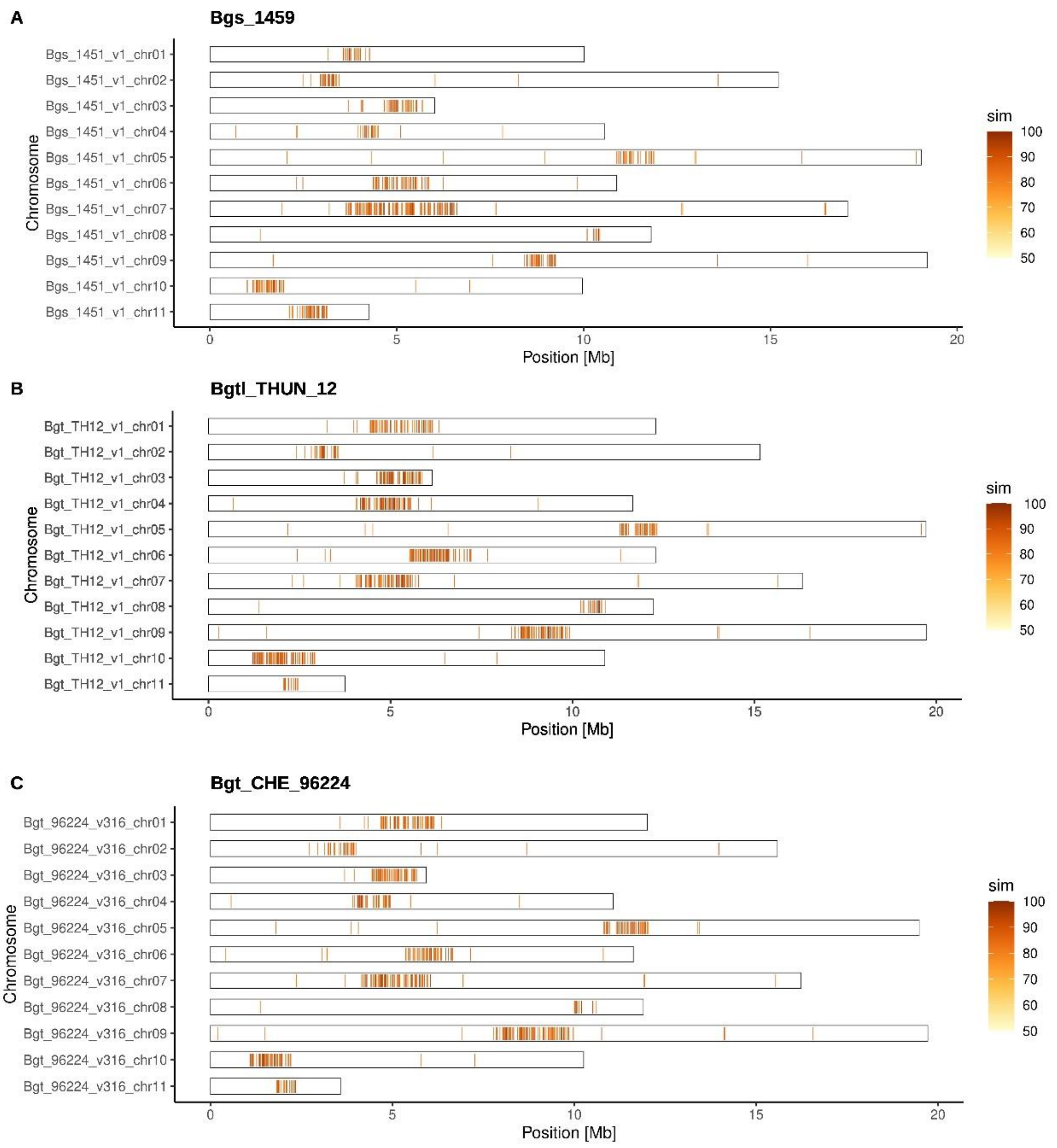

Figure F. Distribution of the LINE *RII_Fuji* in the genomes of three powdery mildews.

Distribution in: (A) The *B.g. secalis* Bgs_1451 genome, (B) the *B.g. triticale* Bgtl_THUN_12 genome, and (C) the *B.g. tritici* Bgt_CHE_96224 genome.

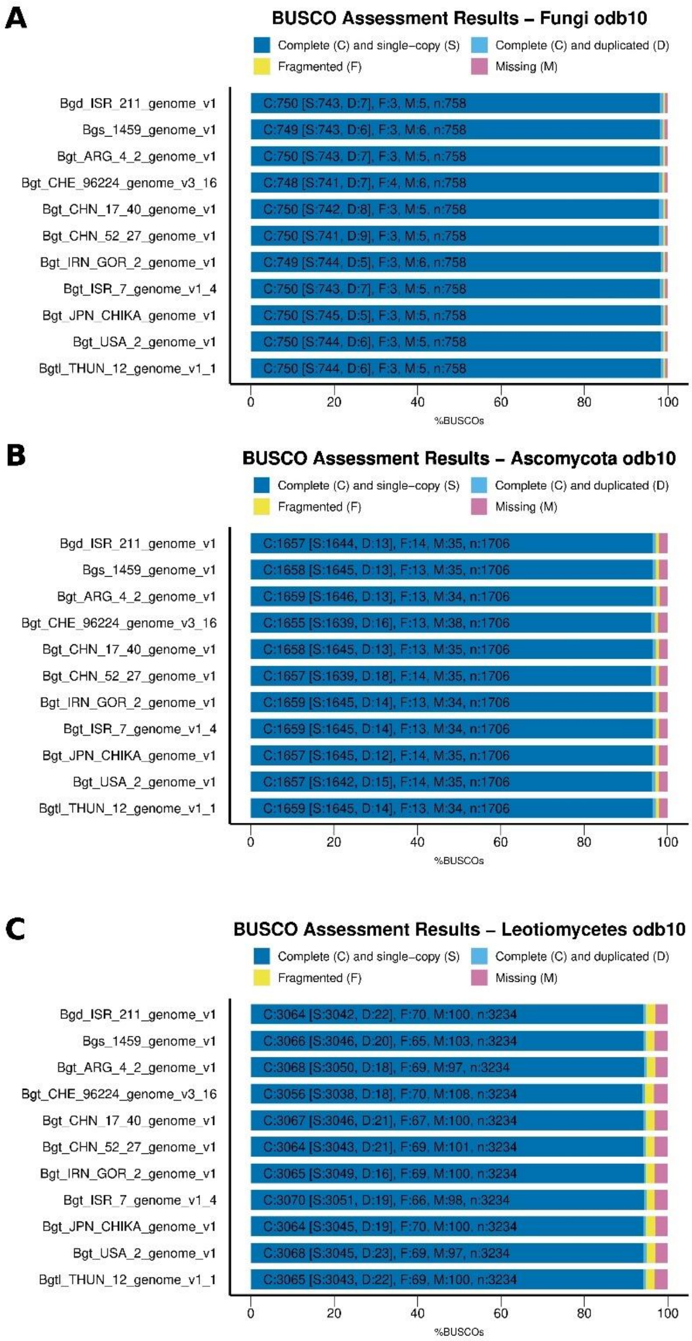

Figure G. BUSCO results of the pangenome.

BUSCO analyses results for all the wheat and rye powdery mildew genomes that have been long read sequenced with PacBio and assembled into chromosomes on three different levels of databases being used: (A) Fungi, (B) Ascomycota, and (C) Leotiomycetes.

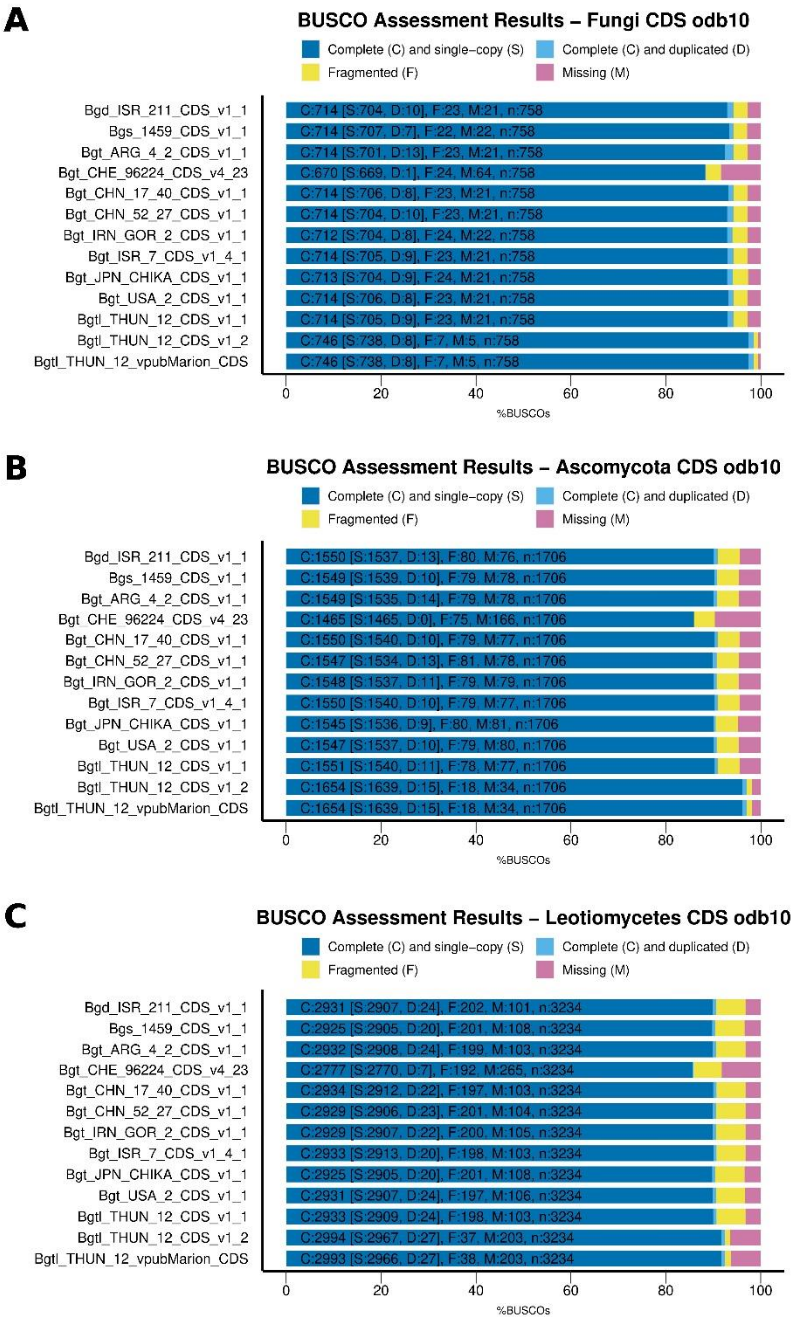

Figure H. BUSCO results of the pangenome's CDS.

BUSCO analyses results for all the wheat and rye powdery mildew coding sequences (CDS) that have been analysed with maker using the near-chromosome scale assemblies, on three different levels of databases being used: (A) Fungi, (B) Ascomycota, and (C) Leotiomycetes.

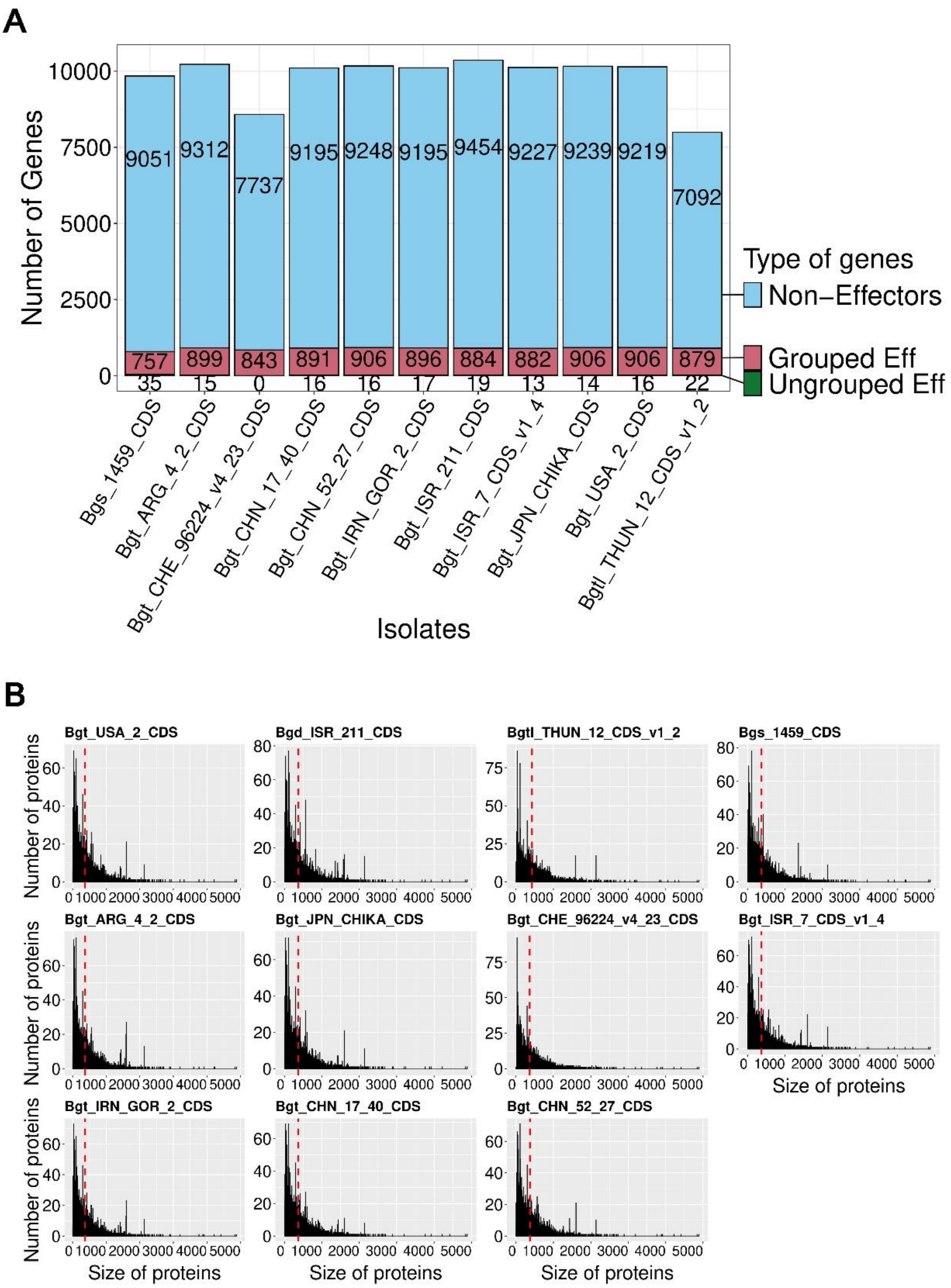

Figure I. Gene annotation statistics.

(A) Number of effector and non-effector genes for all the isolates in the pangenome. Grouped effectors refers to effectors that are homologous to a known effector family, while the ungrouped ones do not belong to a known effector family. (B) Distribution of size of proteins for all the proteins for each isolate.

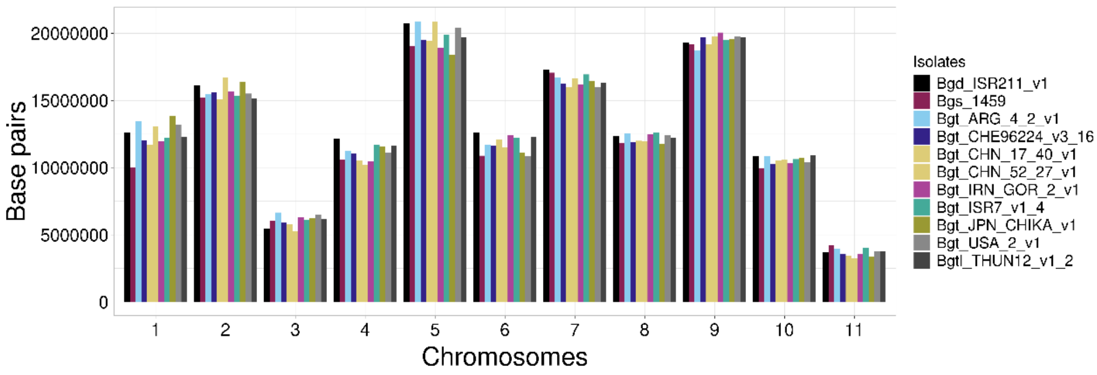

Figure J. Genome statistics and phylogeny of the pangenome.

Chromosomes sizes for all isolates.

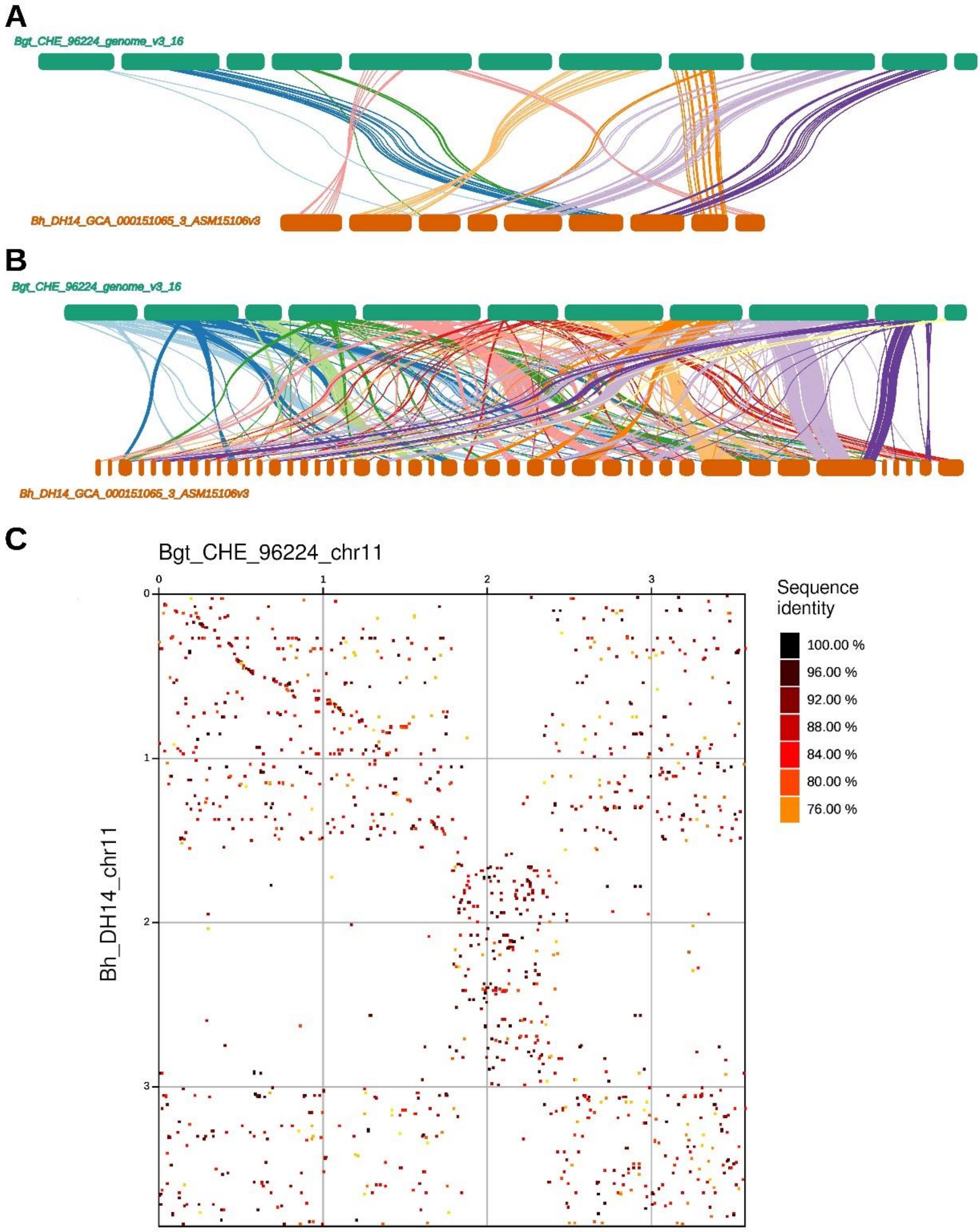

Figure K. Synteny of *B. graminis* and *B. hordei*.
(A) Synteny of *B.g. tritici* Bgt_CHE_96224 (all 11 chromosomes) with the nine largest contigs of *B. hordei* Bh_DH14 using 10kb windows of minimum alignment. (B) Same as (A) with the 100 largest contigs of *B. hordei* Bh_DH14 instead. (C) Dot plot comparison of chromosome 11 from Bgt_CHE_96224 and *B. hordei* Bh_DH14. Chromosome 11 of *B. hordei* was assembled from three sequences contigs (23, 26 and 33) based on their best blast homologies to chr11 of Bgt_CHE_96224, which is why it was not represented in A. Note that sequence homology and collinearity on the left arm is well visible, while it is barely detectable on the right arm.

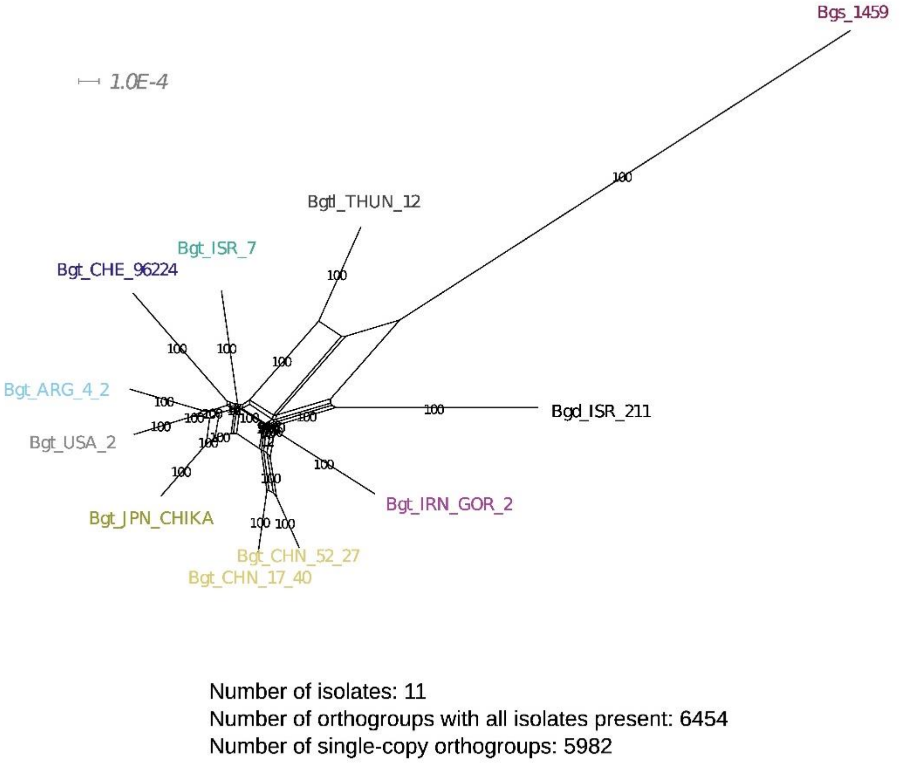

Figure L. Genome statistics and phylogeny of the pangenome.

PhyloNet network on splitstree using single-copy orthogroups via Orthofinder with 100 bootstrap (some of the values are missing from the figure).

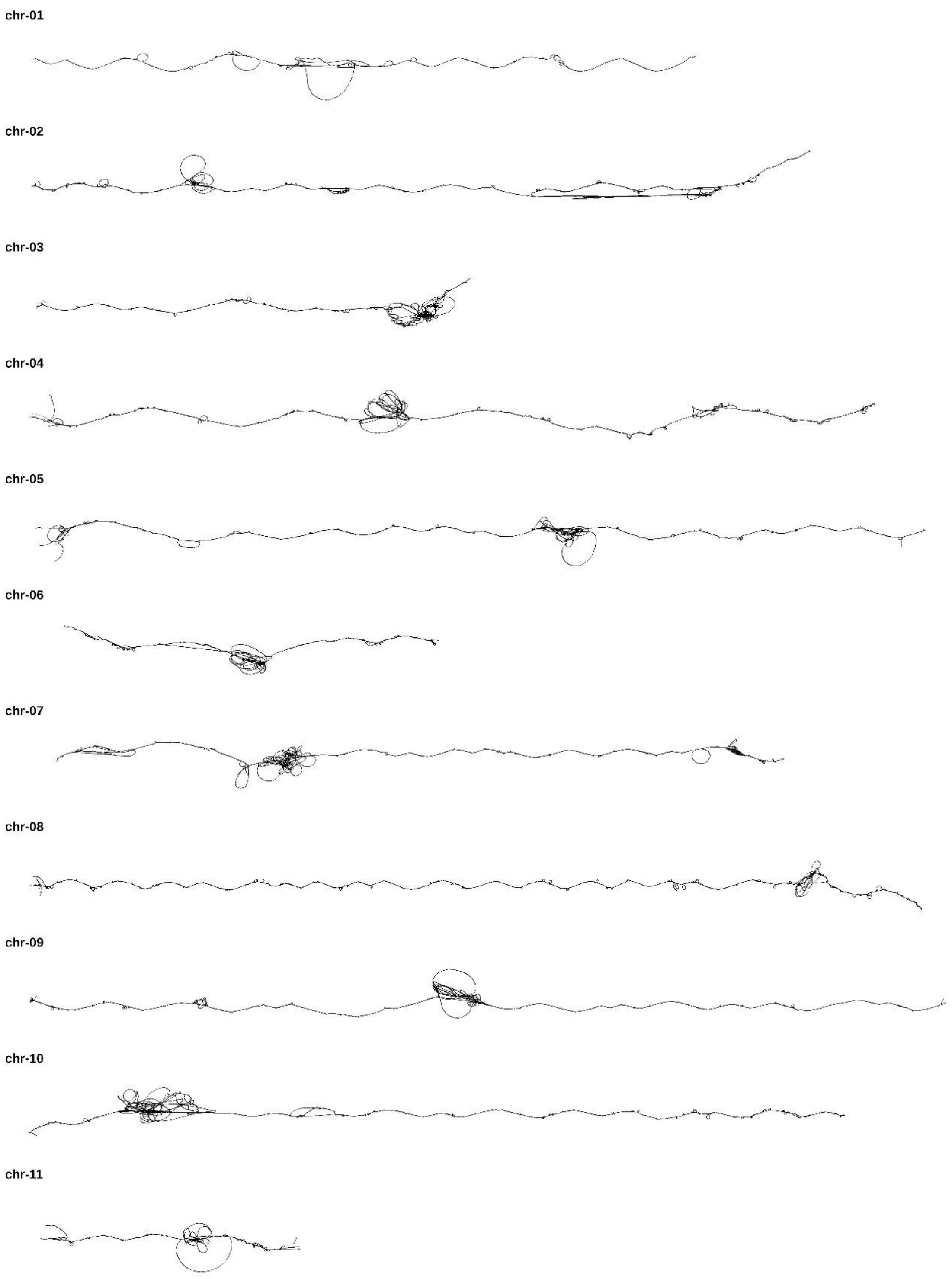

Figure M. Pangenome graph for the 11 isolates included in the *Blumeria graminis* pangenome.

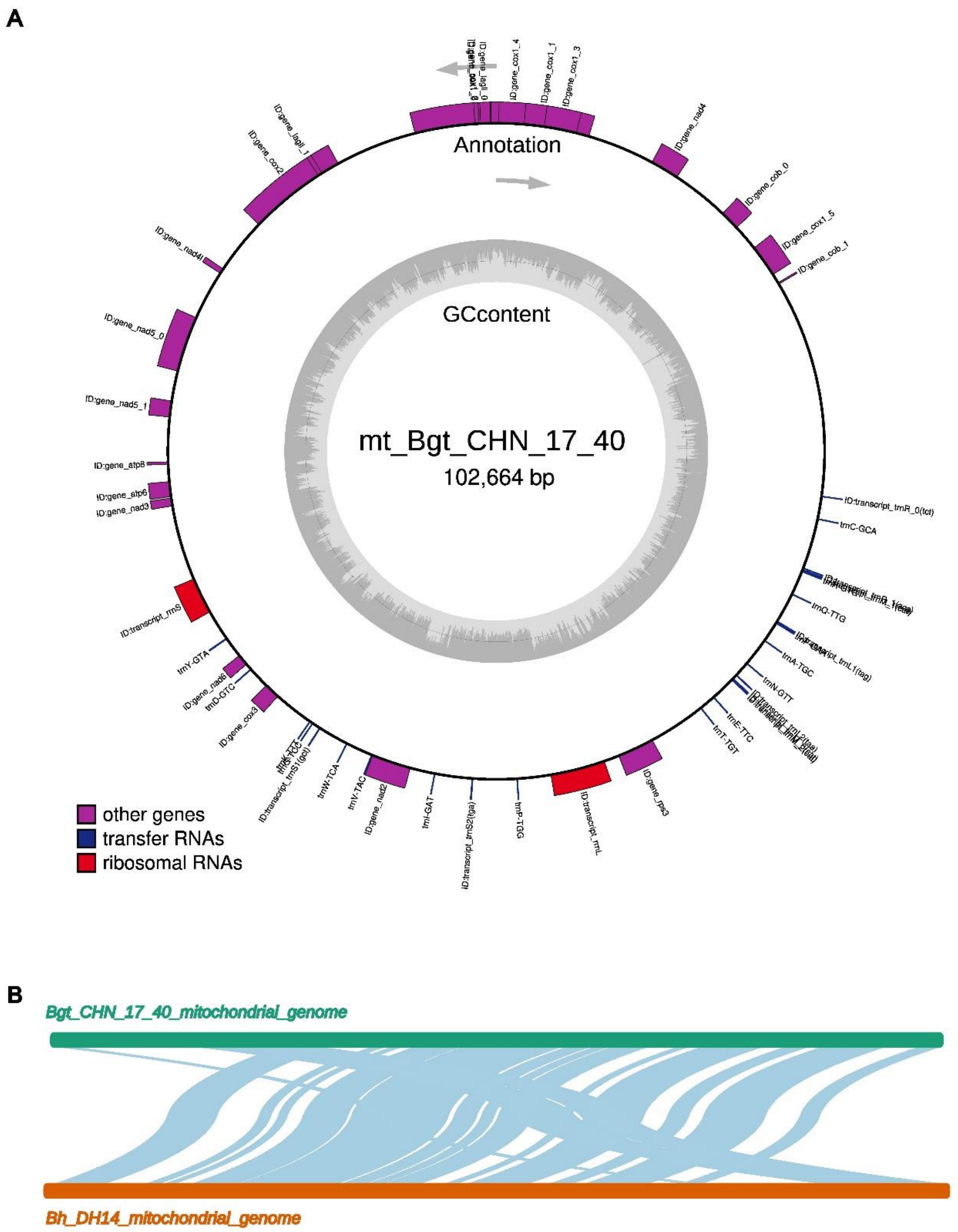

Figure N. Mitochondrial genome visualisation and synteny.
(A) Mitochondrial genome of isolate Bgt_CHN_17_40 along with its annotation of genes etc. The inner circle represents the GC content of the mitochondrial genome. (B) Synteny between the mitochondrial genome of Bgt_CHN_17_40 and Bh_DH14.

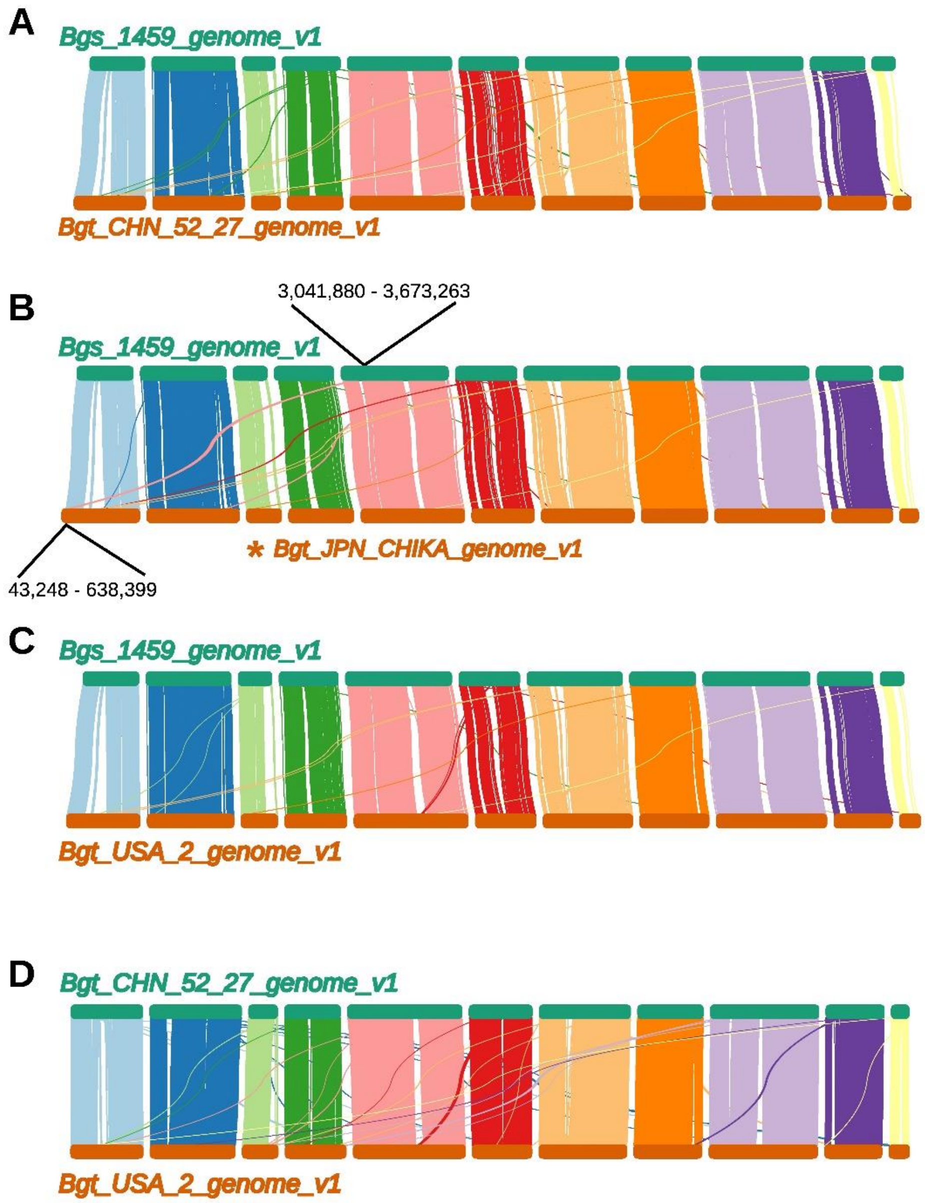

Figure O. Pairs of whole genome synteny for various genomes.
(A) Syntenic pair of the Bgs_1459 and the Bgt_CHN_52_27 genomes in 10kb search windows of synteny, (B) Syntenic pair of the Bgs_1459 and the Bgt_JPN_CHIKA genome in 10kb search windows of synteny, (C) Syntenic pair of the Bgs_1459 and the Bgt_USA_2 genome in 10kb search windows of synteny, (D) Syntenic pair of the Bgt_CHN_52_27 and the Bgt_USA_2 genome in 10kb search windows of synteny. The asterisk represents the genome that is a hybrid one.

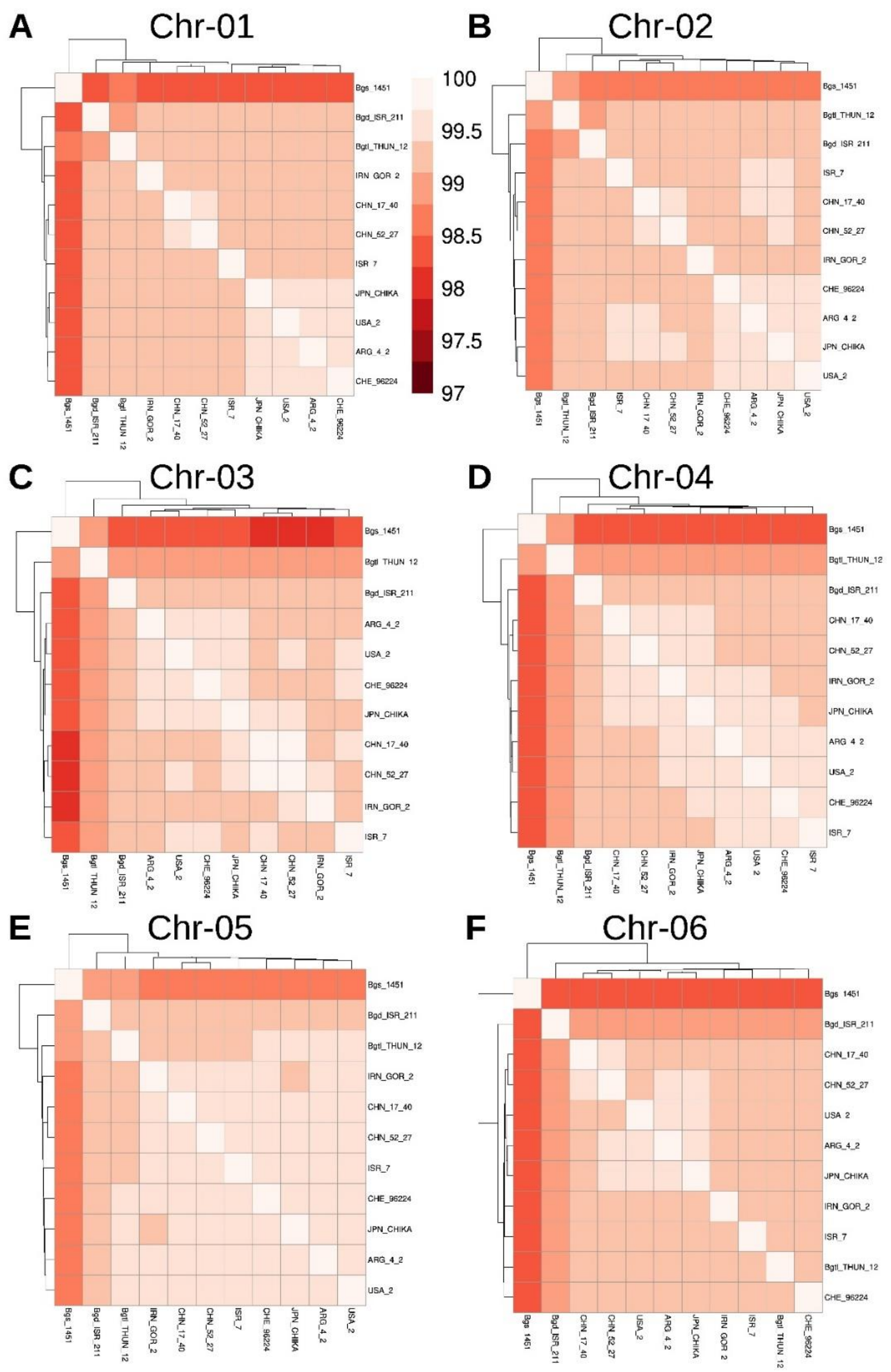

Figure P. Heatmap of independent chromosome average nucleotide identity (ANI) comparisons, using OrthoANIu tool.

(A) Chr-01, (B) Chr-02, (C) Chr-03, (D) Chr-04, (E) Chr-05, (F) Chr-06, (G) Chr-07, (H) Chr-08, (I) Chr-09, (J) Chr-10, (K) Chr-11, (L) Boxplot of the ANI values for chromosome pairs (with – right side and without Bgs – left side) for all chr from 01 to 10, and then only for chr11. A Kruskal-Wallis rank sum test was performed. The p-values show statistically significant difference between chr11 pairs and all the rest of

the chromosomes in pairs ANI values in both cases (including & excluding the Bgs chromosomes).

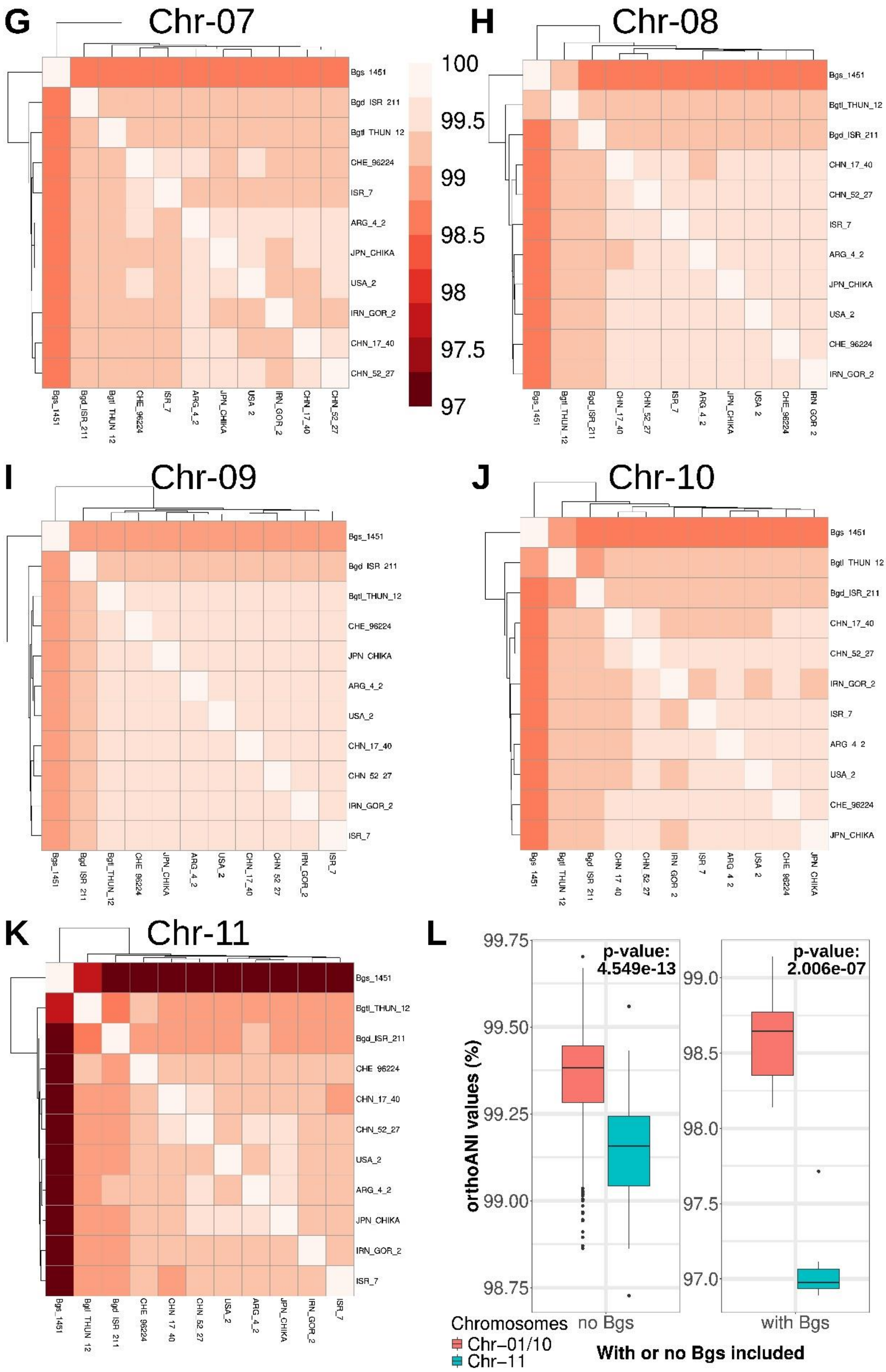

Figure P. Heatmap of independent chromosome average nucleotide identity (ANI) comparisons, using OrthoANIu tool.

(continued)

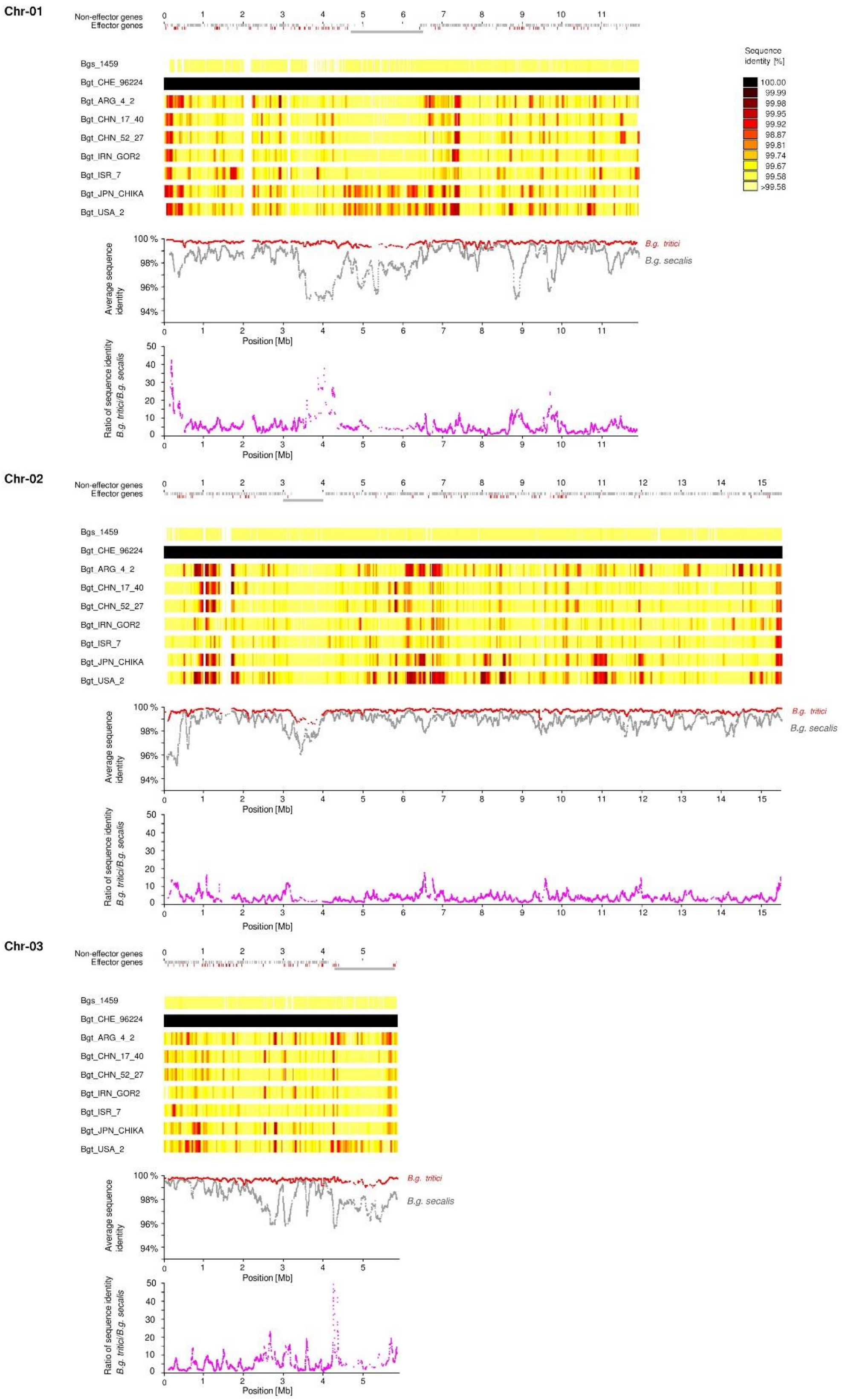

Figure Q. Sequence conservation and diversity along the chromosomes.

Sequence conservation across all chromosomes from chr-01 to chr-11. The heat maps show comparisons of *B. graminis* isolates with the reference isolate Bgt_CHE_96224 in 50 kb windows on the top. The black vertical bars on top signify

the non-effector genes, while the red vertical bars below the black ones signify the effector genes. The red line in the middle shows the average sequence conservation for all 50kb windows among *B.g. tritici* isolates, while the gray line shows sequence conservation between Bgt_CHE_96224 and Bgs_1459. Note that sequence conservation is generally lower in the broader centromere. Also, there are a few small regions in a few chromosomes (chr-01, chr-02), where the sequence conservation is much lower than the rest of the chromosome. The ratio of the sequence conservation between *B.g. tritici* isolates and Bgs_1459 is shown at the bottom of the subfigure in pink.

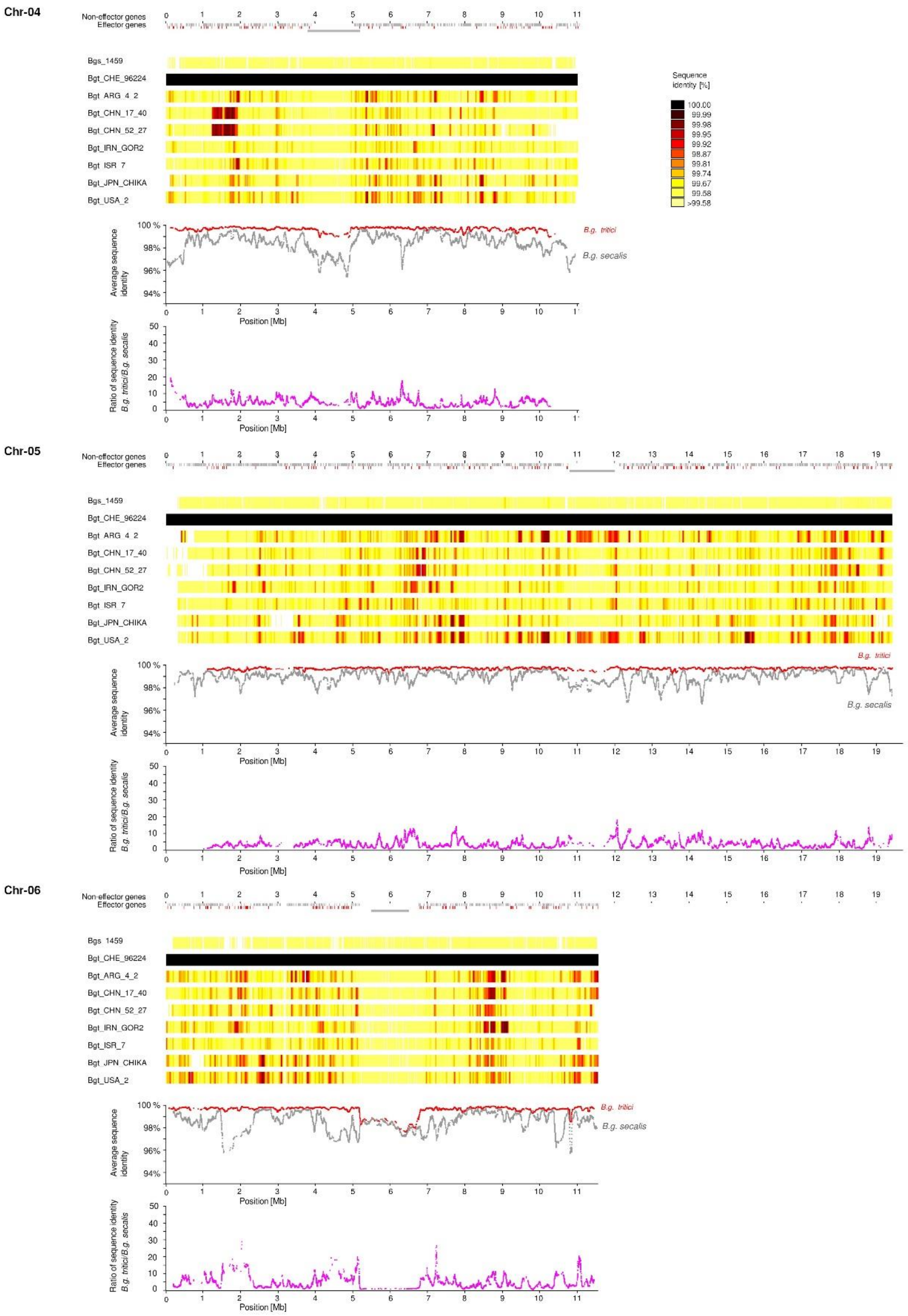

Figure Q. Sequence conservation and diversity along the chromosomes.

(continued)

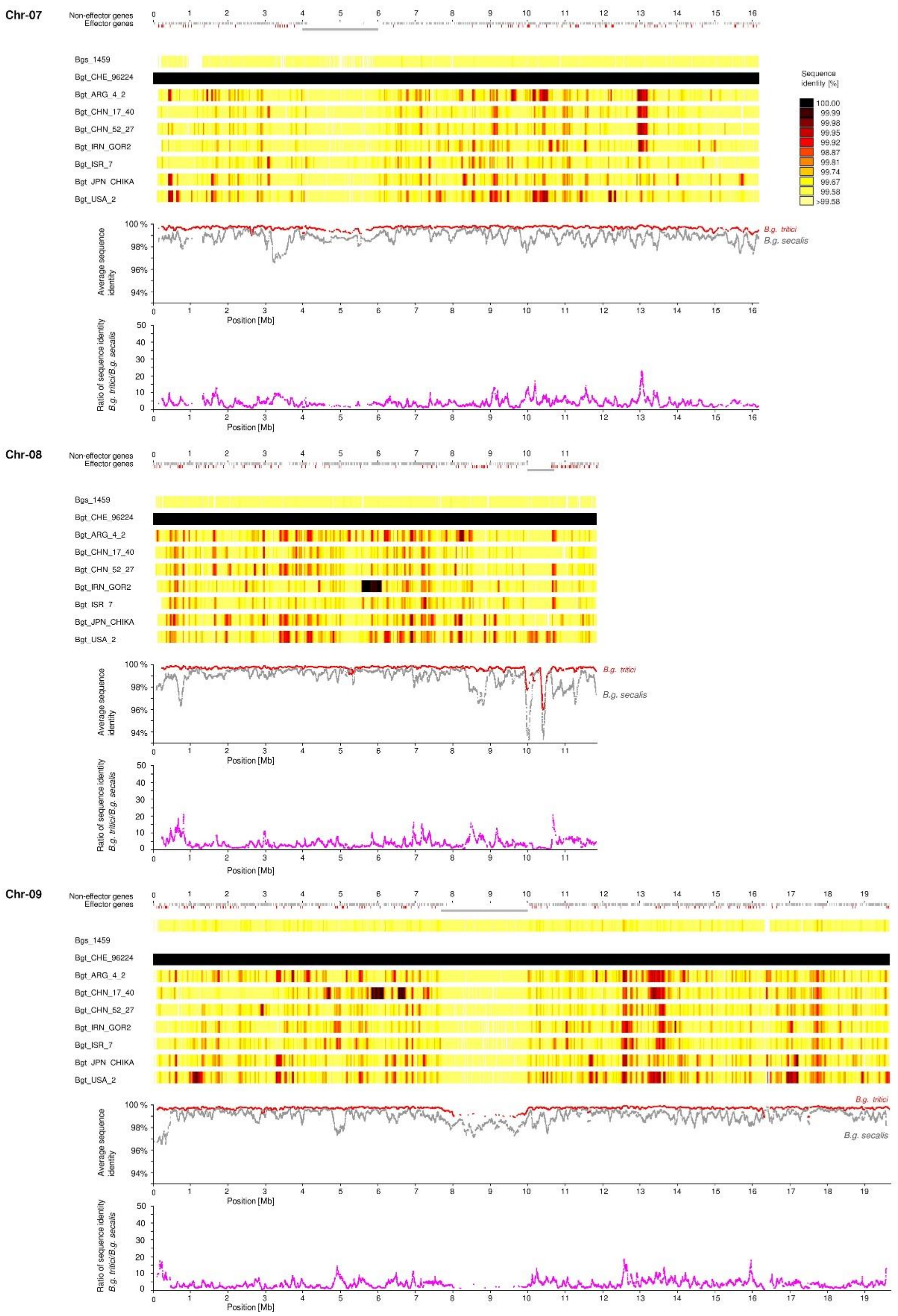

Figure Q. Sequence conservation and diversity along the chromosomes.
(continued)

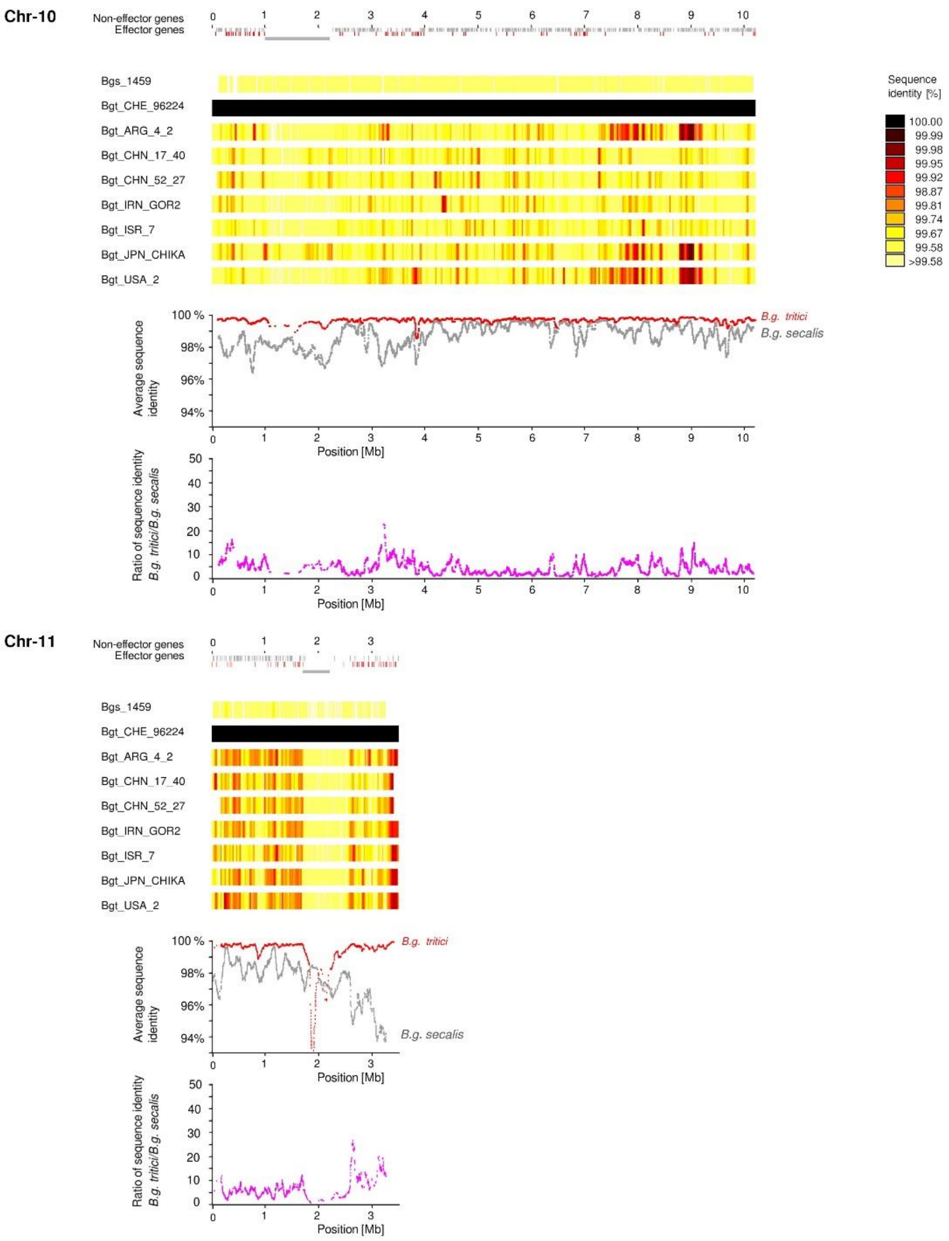

Figure Q. Sequence conservation and diversity along the chromosomes.
(continued)

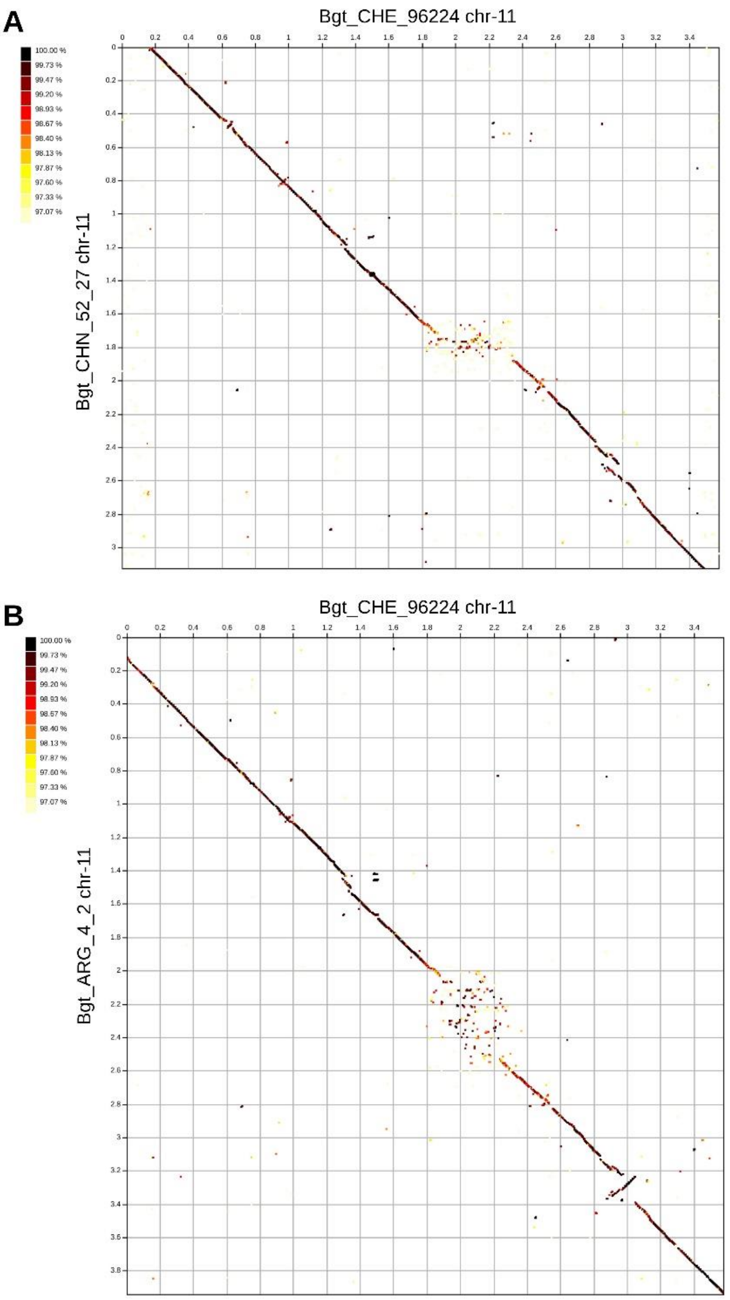

Figure R. Dot plot comparisons of chr-11 of *B. graminis* isolates.

(A) Comparison of chr-11 from the *B.g. tritici* isolates Bgt_CHE_96224 and Bgt_CHN_17_40. Note that Bgt_CHN_17_40 is missing segments of ~180 kb and ~150 kb at the left and right ends, respectively. (B) Comparison of chr-11 form the *B. g. tritici* isolates Bgt_CHE_96224 and Bgt_ARG_4_2. Note that Bgt_ARG_4_2 has an ~150 kb segment at the left end that is not found in Bgt_CHE_96224 and other *B.g. tritici* isolates.

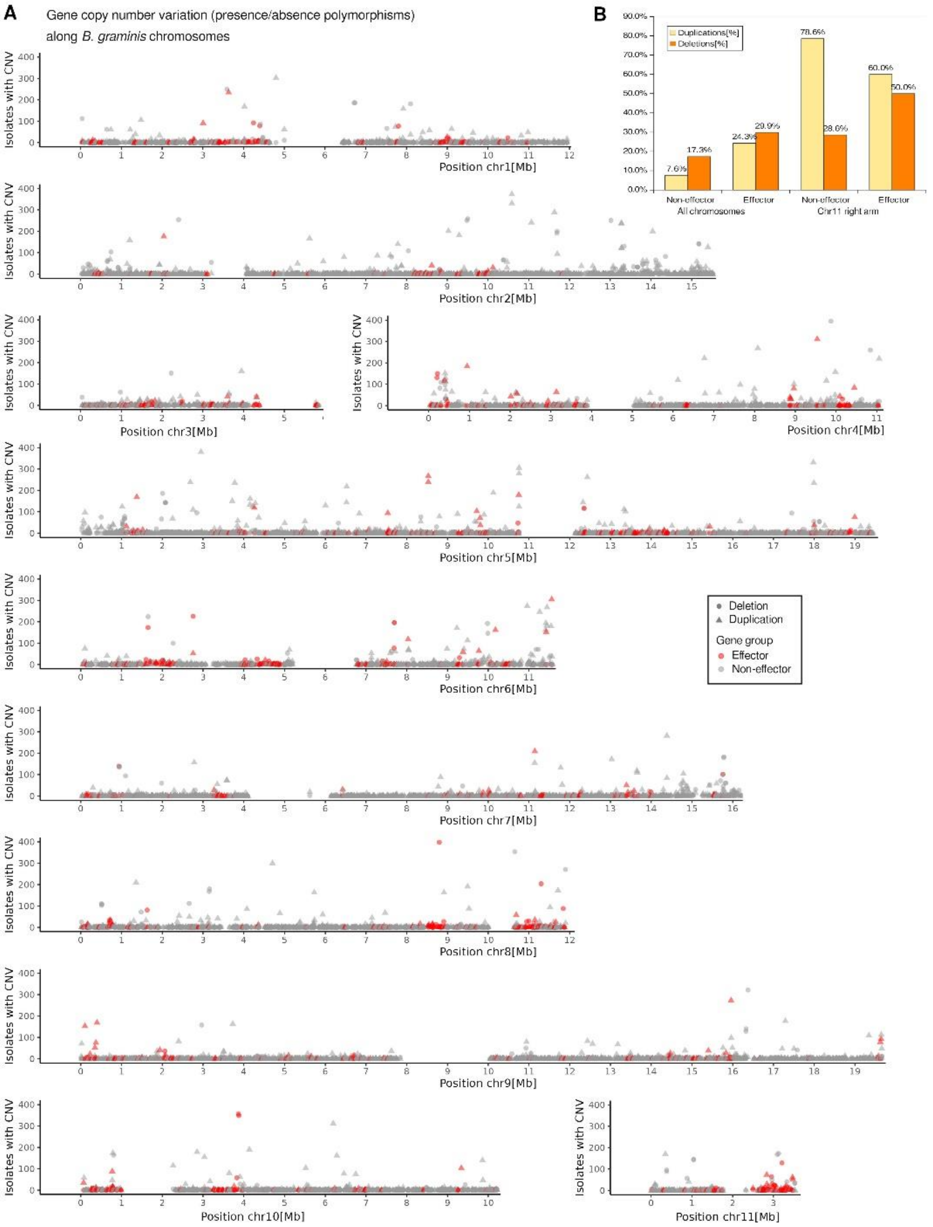

Figure S. Gene presence/absence polymorphisms along *B. graminis* chromosomes detected through sequence coverage with Illumina reads in 399 *B. graminis* isolates.

(A) Copy number variation (CNV) along chromosomes. The x-axis indicates the position in Mb while the y-axis shows the number of *B. graminis* isolates that show copy number variation in a given gene. Circles indicate multiple copies of genes while triangles indicate deletions. Genes were separated into effectors (red) and non-effector genes (gray). Centromeres can be recognized as gene-free regions. (B) Summary of proportions of genes that show CNV. Effectors and non-effectors are shown separately, as are values for all chromosomes and for the right arm of chromosome 11. The y-axis shows the percentage of genes that show duplications and/or deletions in at least 1 isolate. Note that the total of duplications and deletions

can exceed 100% because some genes may be duplicated in one isolate but deleted in another.

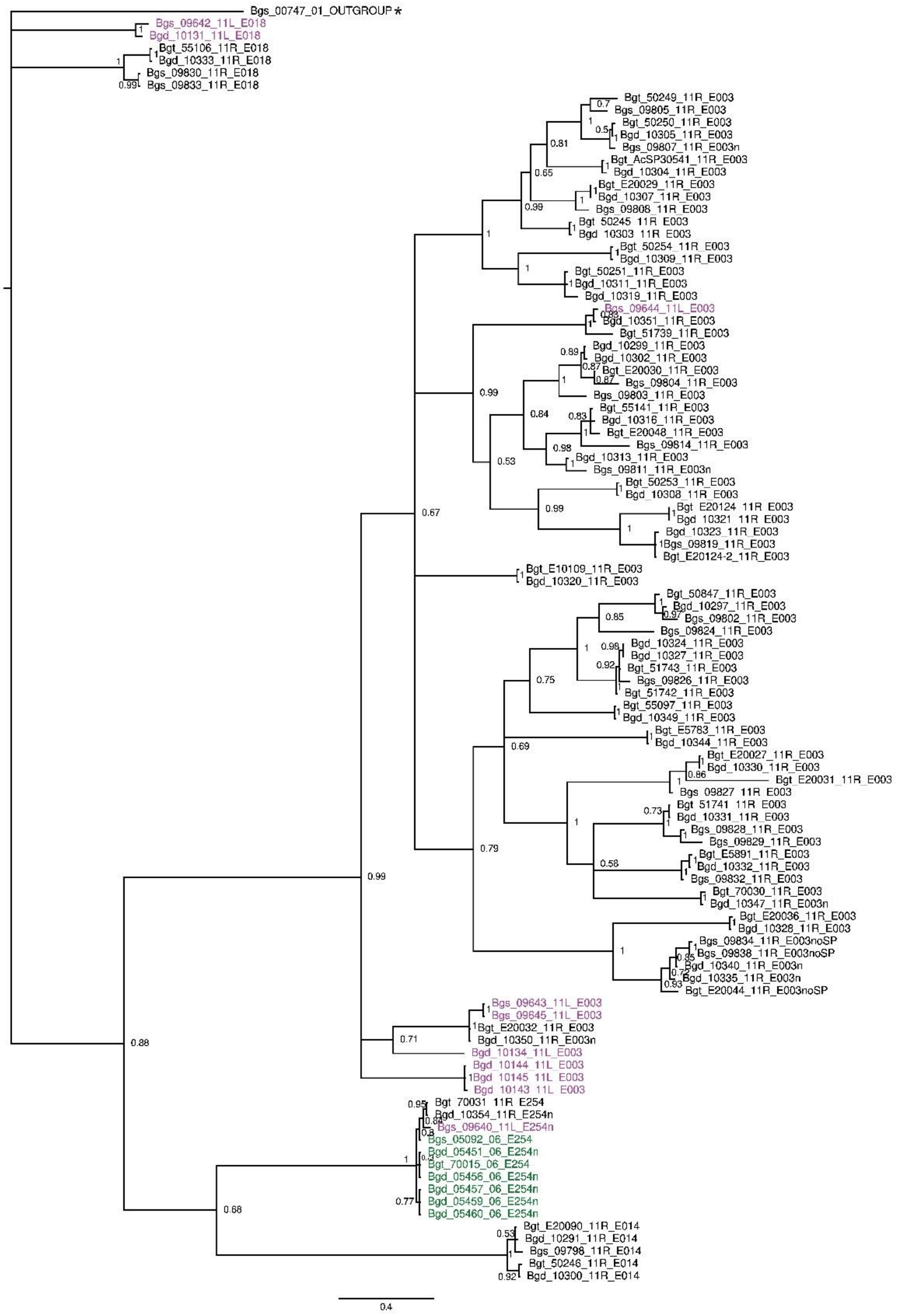

Figure T. Effector protein phylogeny.

Phylogenetic tree of all candidate effector proteins from the right chromosomal arm for Bgs_1459, Bgd_ISR_211, and Bgt_CHE_96224, along with the effectors on the left start region that is missing in the *B.g. tritici* isolates and homologous effectors of Bgs1459-09640 found in chr-06. We used Bayesian inference with MrBayes on ClustalW aligned amino acid sequences of all and the outgroup candidate effector Bgs1459-00747. The effectors in black font colour refer to all the effectors of the right chromosomal arm of chr-11, the ones in red colour refer to all the effectors on the left

start region which is missing in *B.g. tritici* isolates and the ones in green refer to the effectors in chr-06. The outgroup effector has an asterisk on the right side.

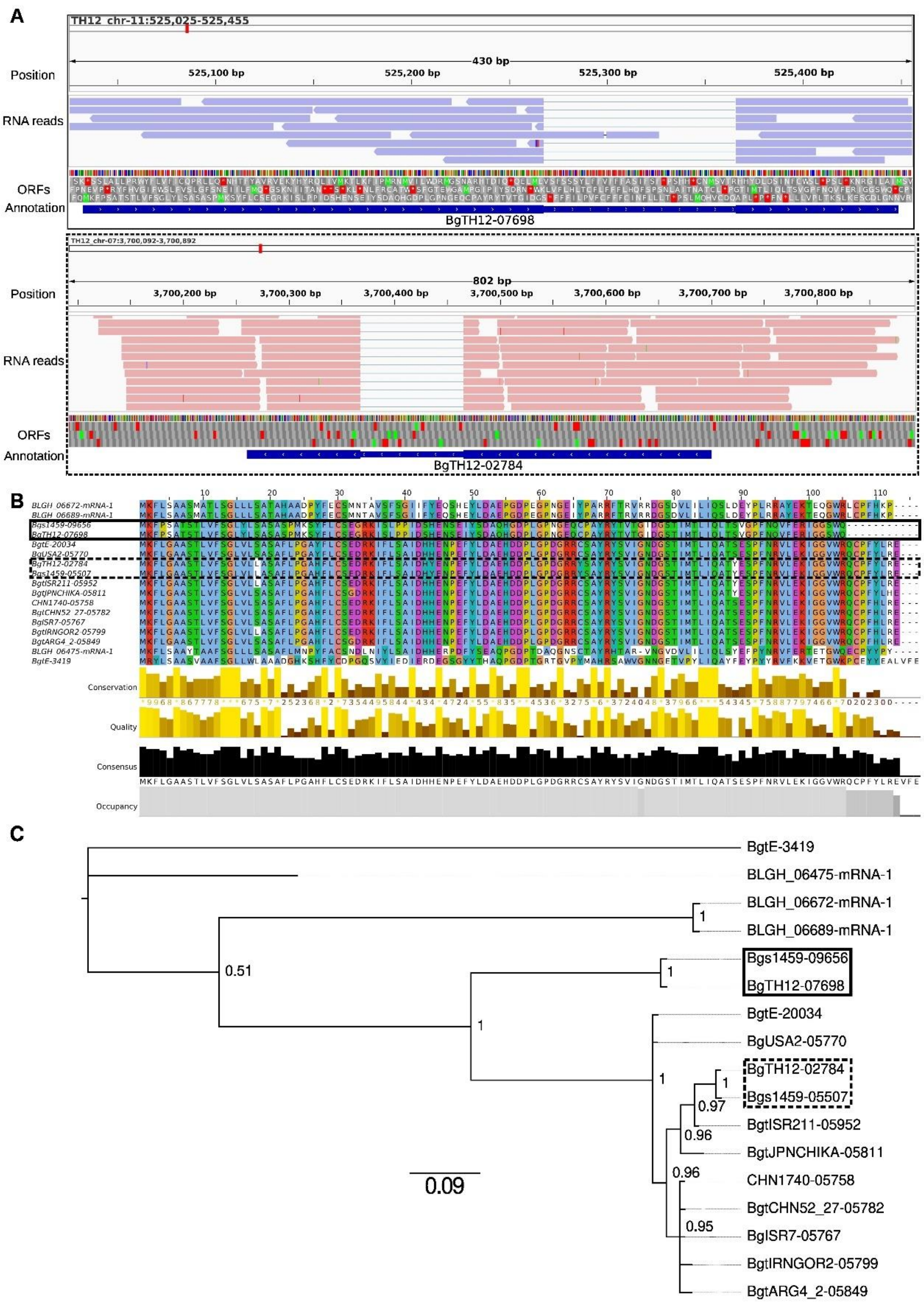

Figure U. Candidate effector comparisons for proteins: BgTH12-07698 and Bgs1459-09656.

(A) Annotation and alignment of RNAseq reads of the BgtI_THUN_12 isolate infecting the triticale culitvar Timbo at two days post inoculation that was used to verify the expression and correct annotation of two candidate effector genes of

interest. (B) Alignment of the unique candidate effectors found only in the *B.g. secalis* and *B.g. triticale* isolates, along with the closest homologous other candidate effector protein found in all isolates in chromosome 7. Cysteines for possible cysteine bridges at positions 30 and 66. (C) Genealogic tree of candidate effector genes that had at least some homology with the candidate effectors above. Most of these proteins belong to the effector family E003. BgtE-20034 protein was used as an outgroup.

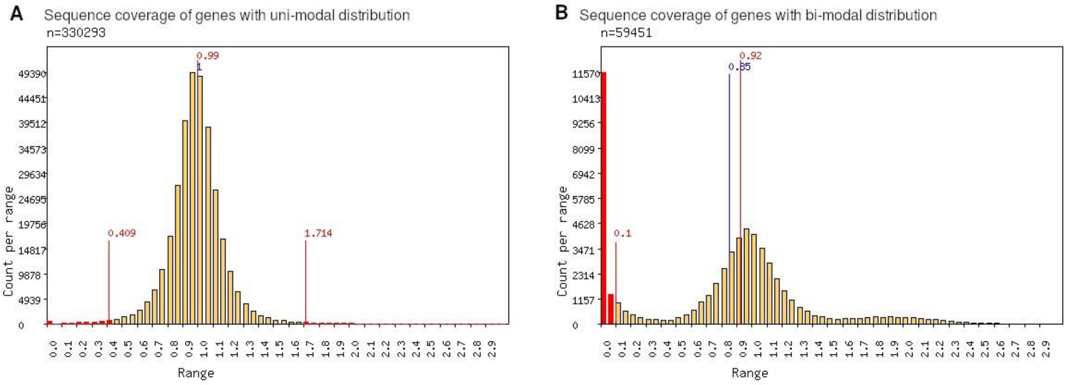

Figure V. Establishment of thresholds to determine gene copy numbers based on Illumina sequence read coverage.

Read coverage was calculated for individual genes across all 399 mildew isolates used in this study. For each gene, it was statistically tested whether reads coverage across the 399 isolates showed uni-modal or bi-modal distribution. (A) Read coverage distribution for genes that showed uni-modal distribution. As threshold for multiple copies, the upper 1 percentile was used (1.7, indicated in red), meaning that 99% genes have read coverage below 1.7. (B) Read coverage distribution for genes that showed bi-modal distribution. Since the group of genes without coverage was very distinct, a value of 0.1 was used (indicated in red), more stringent than the value of the lowest percentile (0.409 shown in A).

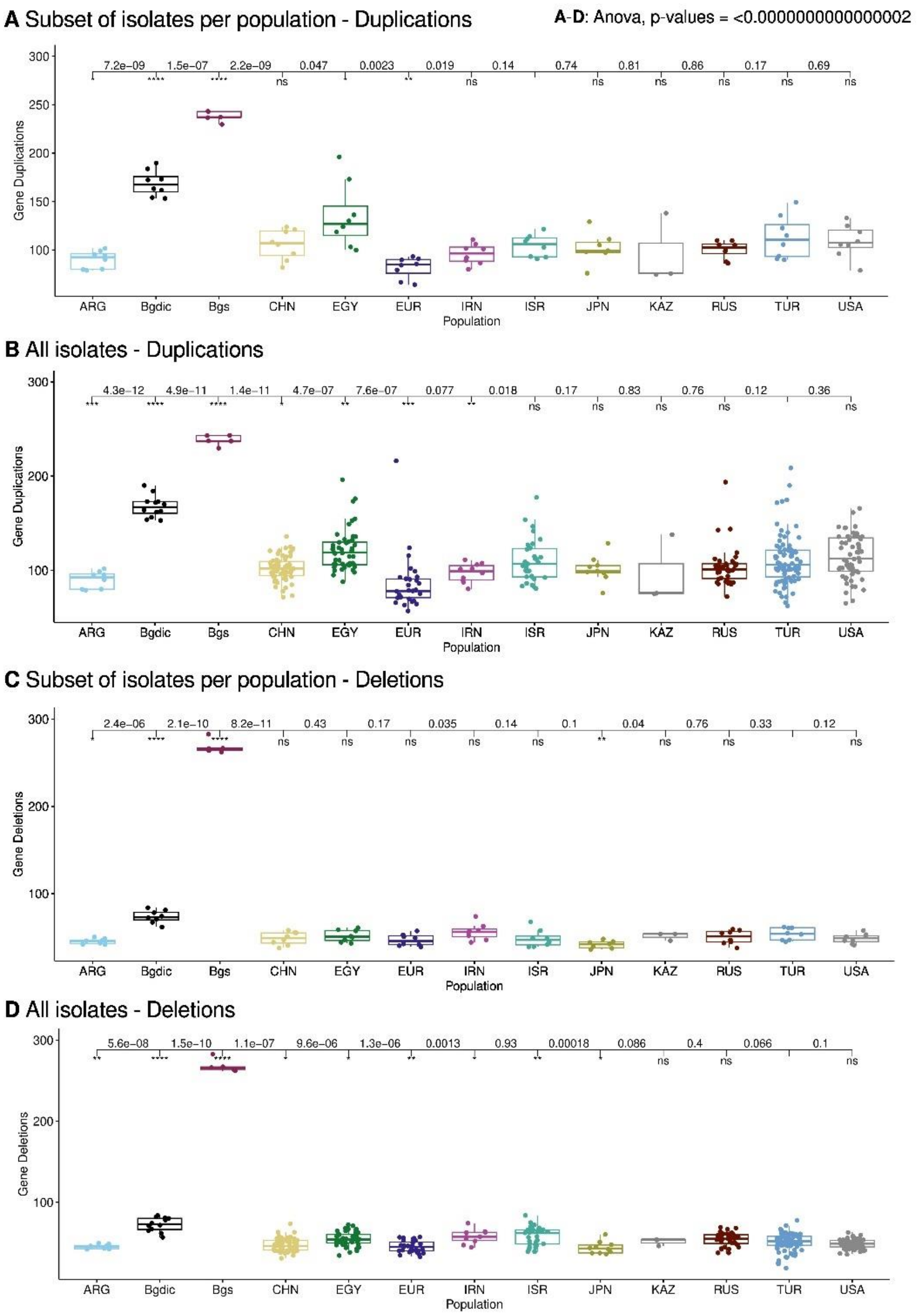

Figure W. Duplications and deletions in various datasets.

Duplications and deletions for all isolates or subsets of isolates per population for most geographic populations and for all genes (see Table B in S2 Appendix). (A) Duplications in subsets of eight randomly chosen isolates per population, (B) Duplications in all isolates per population, (C) Deletions in subsets of eight randomly chosen isolates per population, (D) Deletions in all isolates per population.

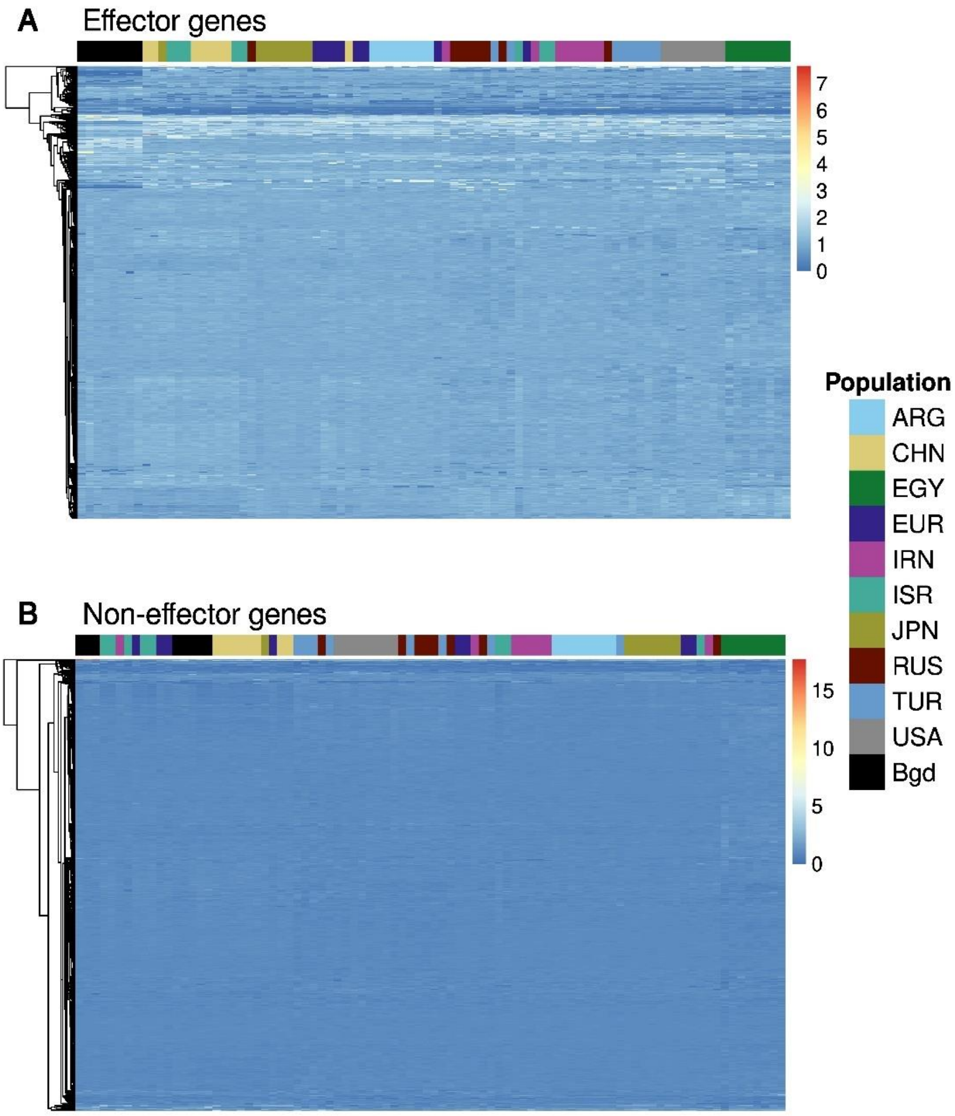

Figure X. Heatmap of CNVs of effector genes in various isolates.

The Bgt_CHE_96224 isolate has a consistent blue colour of medium dark blue for all genes. Darker blue colour than this signifies a possible deletion of a gene, while lighter blue to red colours signifies possible different levels of duplication of a gene. Note that despite its normalization for read coverage and against the reference, many artefacts might still exist due to annotation biases, searching biases etc. Each column refers to one isolate, while each row refers to one gene. (A) refers to the effector genes, while (B) refers to non-effector genes.

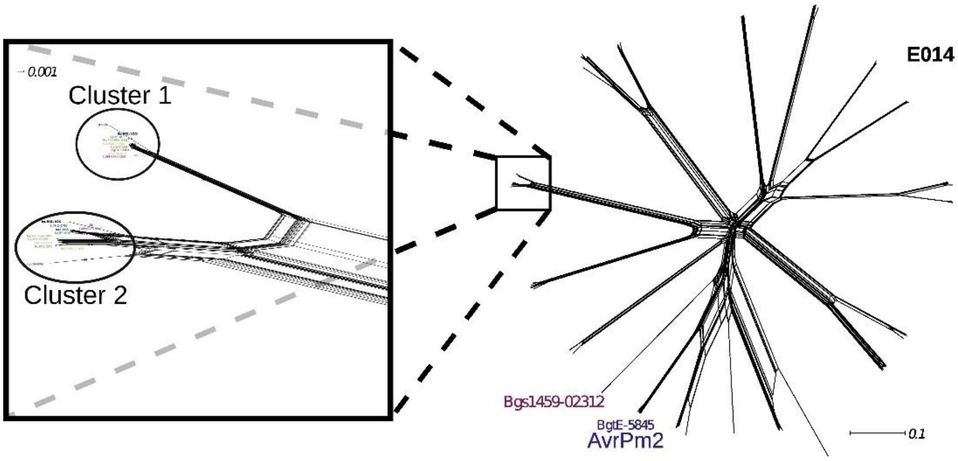

Figure Y. Phylogenetic network of one of the most diverse effector families in wheat powdery mildew (E014).

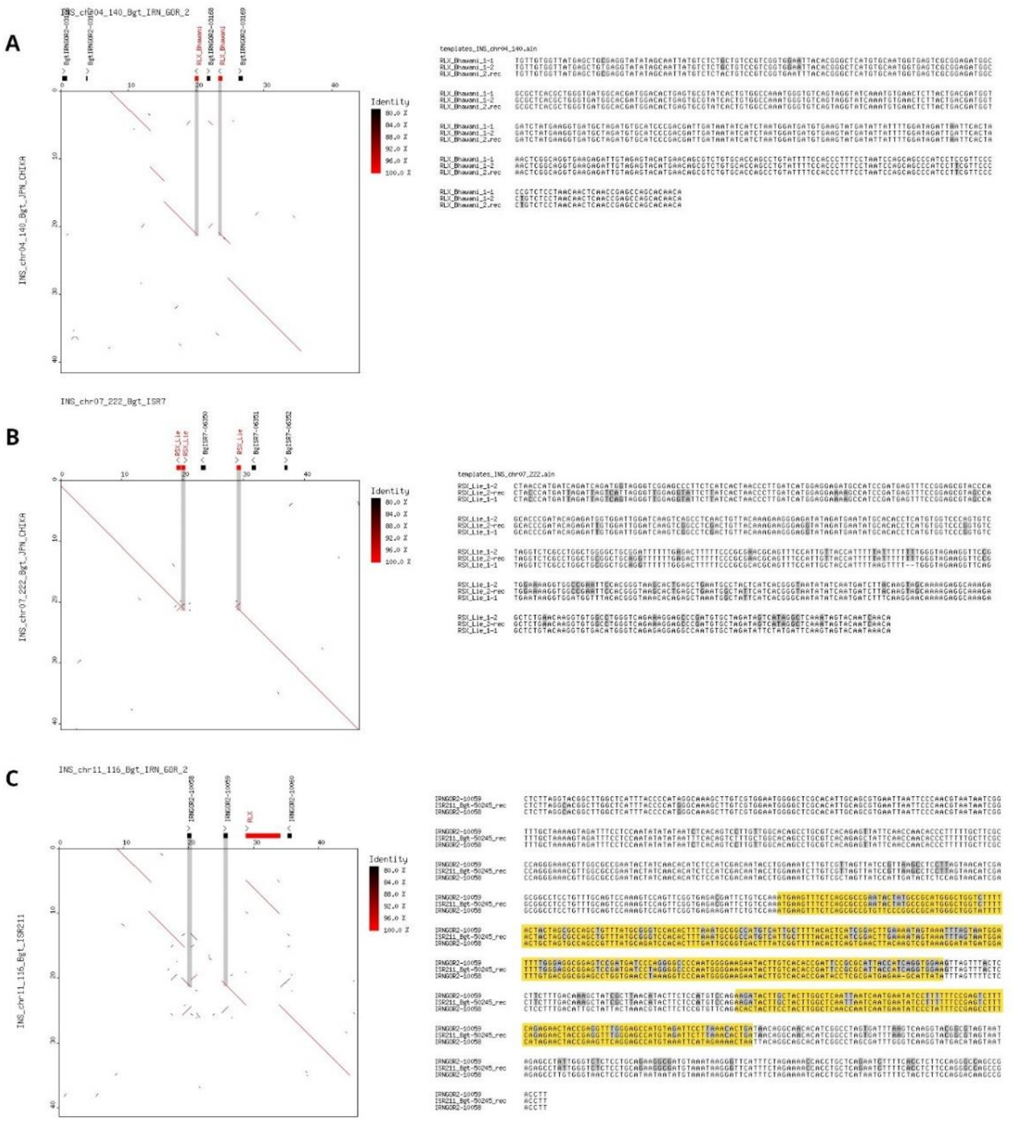

Figure Z. Examples for effector gene presence/absence polymorphisms caused by unequal homologous recombination.

The left side of the panels shows a dot-plot alignment of the region carrying the polymorphism. Gene and TE annotation for the mildew genome that carries additional sequence is shown above the plot with transcriptional orientation indicated by arrow heads. For easier visibility, the TE or gene that served as template for the unequal homologous recombination is connected by shaded areas to the corresponding point in the plot. The right side of the panel shows a multiple alignment of the sequence that serves as template. Recombinant sequences (which is found in the isolate that carries the deletion) has the extension “rec”. Note that the rec sequence, as expected, shares diagnostic SNPs with the left template in the 5’ region and with the right template in the 3’ region, respectively.

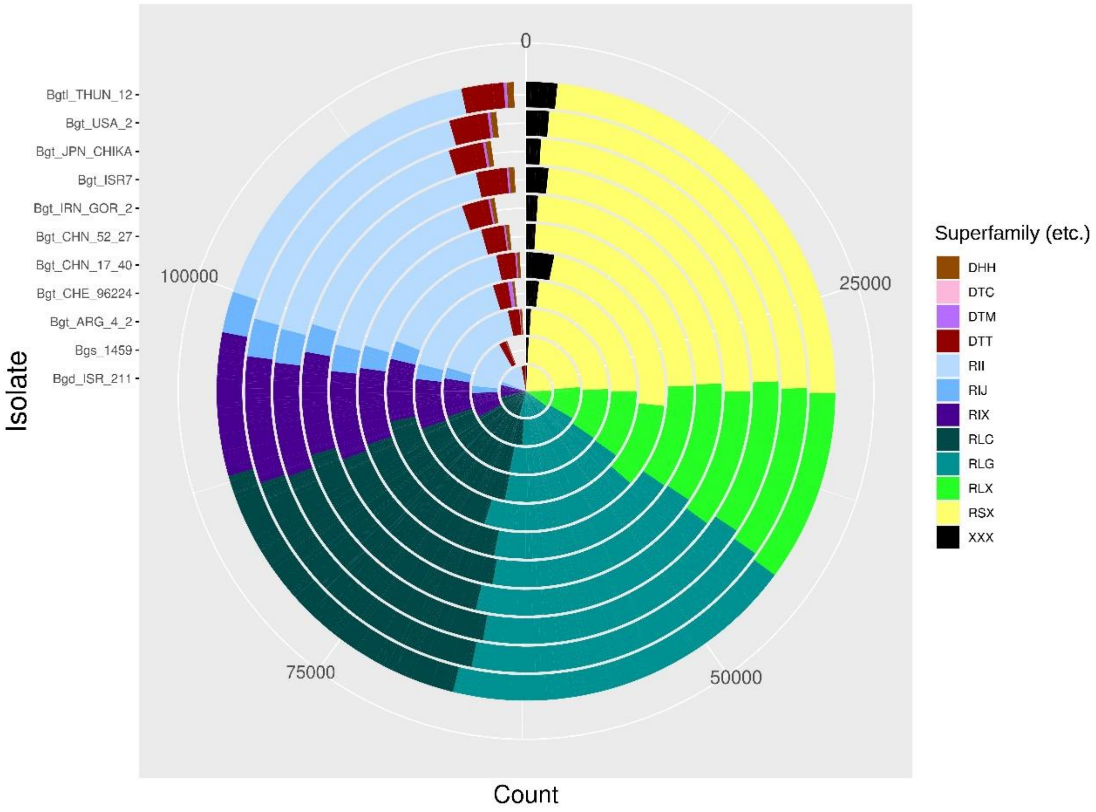

Figure AA. TE analyses in *Blumeria graminis*.

TE counts of TE superfamilies (and other TEs) for the pangenome.

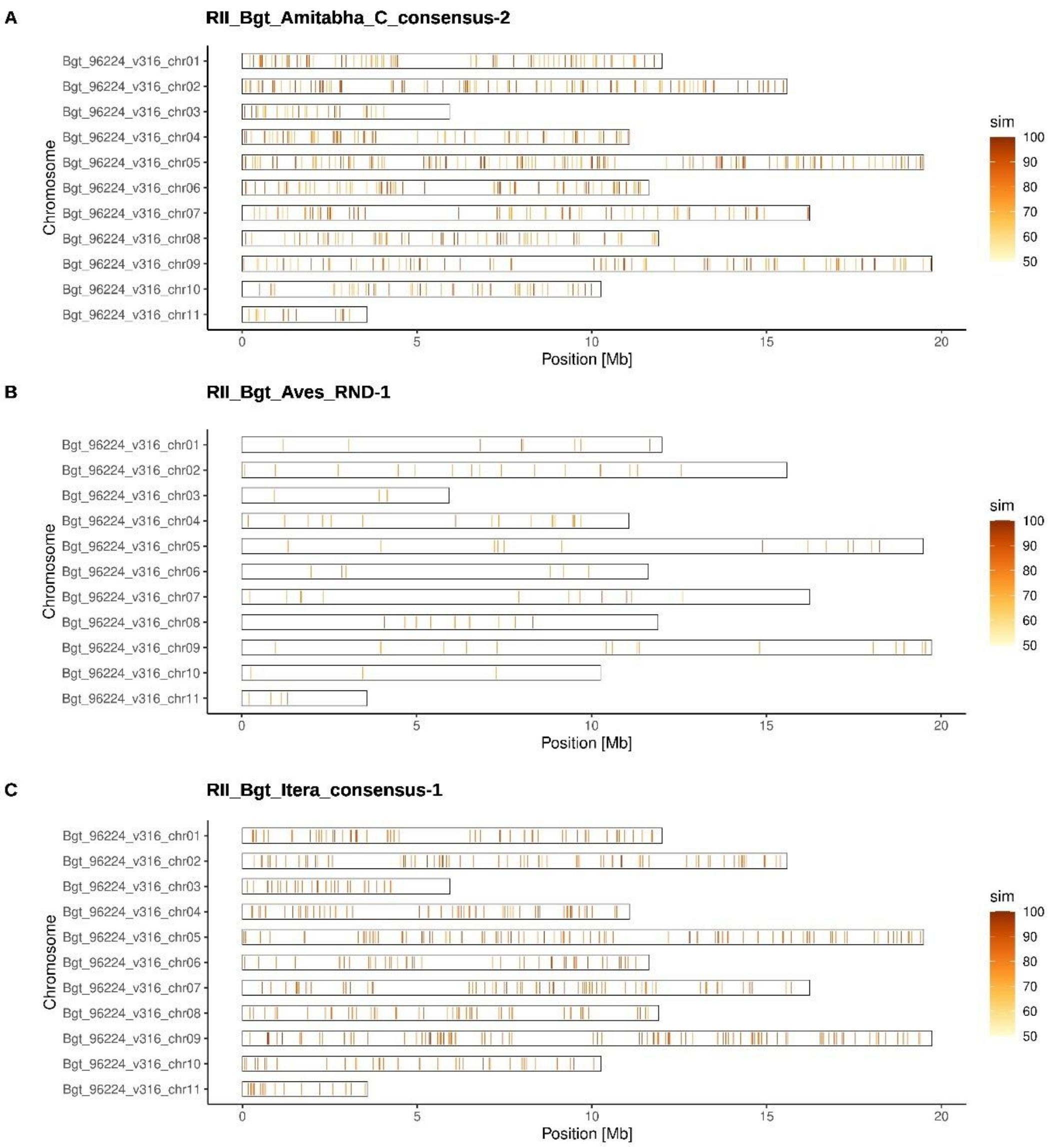

Figure AB. Genome wide distribution of various TE families of interest.

Distribution of various TE families across the Bgt_CHE_96224 genome after a blast with a cutoff of hits >500bp and with 70% identity. (A) *RII_Amitabha_C*, (B) *RII_Aves*, and (C) *RII_Itera*.

Supplementary Tables – Legends

S2 Appendix

Table A. List of isolates used in one or more analyses and details about them. The forma specialis is determined based mostly on the genomic PCA, as in some cases the type of host is not available.

Table B. List of eight random isolates used for multiple analyses (1st column) (randomised using random.org). Singletons results for the random eight isolates per population.

Table C. List of isolates used in the Mantel test analyses. The *B.g. dicocci* isolates are completely excluded from these analyses

Table D. CRAQ (Clipping Reveals Assembly Quality) results for reference-free genome assembly evaluation including the accuracy of assembled genomic sequences for the isolates that were sequenced in this study.

Table E. Statistics of genomes in *Blumeria graminis* pangenome.

Table F. Pangenome statistics including number of nodes, edges, and the total length of the pangenome.

Table G. Whole genome average nucleotide identity (ANI) comparisons, using OrthoANIu tool. Also, mitochondrial genomes are compared, along with genes (CDS).

Table H. Number of genes/ORFs in each mitochondrial genome, under two annotation pipelines.

Table I. Mating types in the *B. graminis* pangenome. We blasted the genomes to look for the mating type genes/loci. *MAT 1-1-1* corresponds to the gene *BgtCHN52_27_00382* in isolate Bgt_CHN_52_27, *MAT 1-2-1* corresponds to the gene *Bgt-3306* and *SLA-2* to the gene *Bgt-2805* in isolate Bgt_CHE_96224.

Table J. Presence or absence of the characterized *AvrPm2* gene *BgtE-5845* coming from Bgt_CHE_96224, along with two other genes of the same effector family in the long-read assemblies of the pangenome isolates. The percentage represents the DNA

homology between the *BgtE-5845* gene that was used as a query in the blast analysis and the assemblies, if no percentage present, then it implies 100% identity.

Table K. TE statistics of the B. graminis pangenome using only copies of identified and annotated TEs, using the genome size, and averaging for the size of identified TEs for the various types (DNA TEs, LINEs, SINEs, LTRs).

Table L. List of isolates and populations used for the detettore analyses.

Table M. Number of copies of the TE RLX_Bgrt_RXB_consenus-1 among the various genomes in the pangenome dataset.

Table N. Statistics of *Blumeria graminis* genome assemblies initial sequencing output.

Table O. Statistics of *Blumeria graminis* genome assemblies after assembly using the software gaas.

Table P. Details on the transposable element identification on the Bgt_CHE_96224 isolate and beyond.
