# Supplementary material for: Combining pangenomics and population genetics finds chromosomal re-arrangements, diversified chromosome segments, copy number variations and transposon polymorphisms in wheat and rye powdery mildew
Source: PLoS Pathog. 2026 Apr 20;22(4):e1013196. doi: 10.1371/journal.ppat.1013196 (PMC13124064; doi:10.1371/journal.ppat.1013196)
Supplement: Attachment — Submitted filename: Response to Reviewers.pdf [file ppat.1013196.s004.pdf]

Response to Reviewers

Reviewers' Comments:

Reviewer's Responses to Questions

Part I - Summary
Please use this section to discuss strengths/weaknesses of study, novelty/significance, general execution and scholarship.

Reviewer Comment 1.1:

Reviewer #1: The authors conducted a comparative genomics and pangenome analysis involving eight hexaploid wheat powdery mildew (Blumeria graminis f.sp. tritici), a tetraploid wheat powdery mildew, a species specialized to infect rye and one for triticale. They provide seven new high-quality genome assemblies and included published genomes as well as sequencing datasets of 399 isolates. Their analysis reveals chromosomal rearrangements within several of these species and/or isolates and highlights a near-accessory quality of chr-11, which was previously unseen for powdery mildew fungi. They further enriched the analysis and found extensive copy number variation among effector groups as well as signs of recent transposable element activity, evidence for which have previously been presented for both wheat and barley powdery mildew. Their work is the first pangenomic analysis of powdery mildew fungi and as such relevant, since such analysis are challenging with obligate biotrophic plant pathogens.

Our response:

We thank reviewer 1 for their constructive comments. We revised the whole manuscript in response to the comments which are addressed point-by-point below (p.4, pp.13ff).

Reviewer Comment 2.1:

Reviewer #2: This study by Sotiropoulos et al. presents a valuable set of 11 high-quality Blumeria graminis genome assemblies from globally distributed isolates, offering a useful resource for fungal genomics and plant-pathogen interaction research. While the sequencing and assembly work is technically strong, the manuscript would benefit from clearer terminology and structure, particularly regarding the use of the term “pangenome,” which is not supported by formal construction of a pangenome graph.

Our response:

We thank the reviewer for the constructive comments. The manuscript was changed now with clearer terminology and structure and substantially added to results and discussion. We also constructed a pangenome graph and did additional analyses on gene presence/absence polymorphisms. We describe the changes and additional analyses in more detail in our responses to reviewers' comments below.

Reviewer Comment 2.2:

Key interpretations, such as the classification of chr11 as "accessory-like" and the analysis of TE content, could benefit from more cautious framing and deeper analysis.

Our response:

We understand the concerns of the reviewer that the term "accessory" is typically reserved of clear presence/absence of entire chromosomes. To address the comment, we have done additional analysis which is described in more detail below (see comment 2.8, p.7). We have now also toned down the language as suggested throughout the manuscript.

Reviewer Comment 2.3:

The reuse of previously published data without clearly highlighting new insights also reduces clarity. Nonetheless, the data generated are of high value, and with improved organization, clearer distinctions between reused and novel findings, and more rigorous comparative analyses, the study could significantly contribute to the understanding of genome evolution in this species complex.

Our response:

We have now highlighted new insights we gained from previously published data by adding more analyses (i.e. admixture, see below) to improve clarity. We have restructured the text throughout the manuscript by shortening/condensing previous findings and expanding the novel findings.

Reviewer Comment 3.1:

Reviewer #3: This large-scale study uses a combination of long-read whole genome sequencing from 9 Pgt isolates and two additional isolates specialized on other hosts as well as population level and short read sequencing of 399

isolates to explore the pangenome of this important obligate biotroph. This work has one of the largest number of isolates sequenced (~400) second only to Z. tritici (1000). Using these data the authors explore whole genome synteny, within population diversity, TE diversity and insertion polymorphisms. While I think that the data and analysis supporting this work is very strong, I found that overall, main emphasis in the results and discussion simply reinforces prior studies on this species complex (i.e. populations are more diverse at the centre of origin and TE insertions are also greater). I feel that the text could be substantially revised to place more emphasis on the new insights that these long-read genomes brought to this important species complex and how the population level data can be used to re-enforce the findings from these long-read assemblies (i.e. effector gene copy number variation in the accessory chr11, can you show the duplicated genes in the context of the chromosome?

Our response:

We have restructured the text throughout the manuscript by shortening/condensing previous finding and expanding the novel findings. Also, in response to Reviewer comments 2.2, p.2 and 2.8, p.7, we show now duplicated and deleted genes in the context of the chromosome (Fig 5B and S18 Fig) and describe it in the text (Line 499ff).

Reviewer Comment 3.2:

And then validate/show variation for a large number of isolates from that same population?).

Our response:

In our analysis of 399 isolates, we found that relatively few gene deletions or duplications are fixed in individual populations with *B.g. secalis* (unsurprisingly) showing the highest number with ~10% of the CNVs being completely fixed. We describe this now in the results (Lines 570-573).

We also checked the fixation rates of the 14 manually analyzed loci that contain presence/absence polymorphisms. Interestingly, we only found high fixation levels for one of the presence/absence polymorphisms in the different populations. This could indicate that unequal recombination might be recurring independently multiple times. We mention this now in the results (Line 632ff) and discussion (Line 841ff).

Reviewer Comment 3.3:

I note the large number of supplemental figures and tables. Unfortunately, as a reviewer I could not access the supplementary tables. Given the large amount of data generated I would encourage the authors to submit these tables-data this to Zenodo to increase accessibility.

Our response:

These tables/data have now been submitted to Zenodo to increase accessibility (see also response to Reviewer Comment 3.15, p.12) (see Zenodo link at the last page 30). We also submit them with the manuscript.

Reviewer Comment 3.4:

My comments below highlight interesting aspects of the work, which I feel could be further clarified or expanded upon to emphasise some of the novel findings of this work.

Our response:

Below we answer the comments point-by-point (responses to Reviewer Comment 3.5, p.8, and following).

Part II – Major Issues: Key Experiments Required for Acceptance

Please use this section to detail the key new experiments or modifications of existing experiments that should be absolutely required to validate study conclusions.

Generally, there should be no more than 3 such required experiments or major modifications for a "Major Revision" recommendation. If more than 3 experiments are necessary to validate the study conclusions, then you are encouraged to recommend "Reject".

Reviewer #1: (No Response)

No comments for “Part II – Major issues”.

Reviewer Comment 2.4:

Reviewer #2: Major Comments

1. Definition and use of the pangenome concept

The manuscript would benefit from a more precise and accurate definition of the term “pangenome.” The authors use this term to describe a collection of reference genomes, but they do not construct a pangenome graph. A true pangenome typically involves a structured representation (e.g., a gene presence/absence matrix or a graph-based approach) that systematically captures shared and variable genomic content across strains. The manuscript should consistently differentiate between the comparison of multi-reference genomes and an actual pangenome. I recommend revising the terminology throughout the manuscript to reflect this distinction.

Our response:

We have now revised the terminology throughout the manuscript to differentiate between the comparison of multi-reference genomes and an actual pangenome. As suggested, we also constructed a pangenome graph which is shown in S13 Fig, discussed in the text (Lines 306-312), and with examples also shown in the new Fig 5A (see also Reviewer comments 2.1, pp.1ff, 2.21, pp.21ff).

However, we did not produce a presence/absence matrix for all genes across the 11 PacBio genomes. As we describe below (see also Reviewer comments 3.5, p.8 and 3.7, p.9), we manually analyzed numerous loci which show gene presence/absence polymorphisms. The results are described in a new paragraph (Lines 617ff), a new Fig 7 and a supplementary figure S24. This analysis required time-consuming and detailed work to sufficiently validate all presence/absence cases, especially in such a highly repetitive genome. We are afraid that an automated approach would not yield reliable enough results.

Reviewer Comment 2.5:

2. Structure and flow of the manuscript

The manuscript currently lacks clear structure and logical flow, which makes it difficult to follow. It is often unclear what constitutes novel findings versus what has been confirmed or re-analyzed from previous work. Greater effort is needed throughout to clearly delineate new data from reused material.

Our response:

We have now more clearly distinguished the new data from the reused material in the entirety of the text (see also responses below).

Reviewer Comment 2.6:
3. New resources data
The first results section titled “New resources for the worldwide study of wheat powdery mildew” reuses published population data and confirms previously reported findings. This is not a problem per se but I struggled to get the novelty. If the aim is to justify the selection of strains for long-read sequencing, this could be presented more concisely as rationale rather than as a primary result. This section could be moved to the supplementary material (with Figure 1 as Figure S1). If the authors wish to highlight new insights into population structure, they should consider complementing the PCA with an ADMIXTURE analysis. This could clarify whether the Central European, Middle Eastern, and Russian samples form a single cluster or exhibit more nuanced structure.
Our response:

We have now placed this finding in the supplementary material section and have re-framed it in the main text. We have highlighted new insights by adding an ADMIXTURE analysis complementing the PCA (shown in Fig. 1C, S2 Fig, S3 Fig and in the results section Lines 196-213, and in the methods in Lines 943-947).

Reviewer Comment 2.7:
4. Mitochondrial genome assembly
Is the mitochondrial genome of Bgt_CHN the only one that is fully assembled? What was the rationale for including the mitochondrial genome + unassembled contigs as chr_Un in the other assemblies instead of extracting the mitochondrial genome directly using Bgt_CHN as a reference? This needs clarification, as the current approach appears inconsistent. In addition, I would prefer to retain the unassembled contigs as they are, rather than attempting to artificially assemble them into a chimeric 'chromosome' that lacks biological meaning.
Our response:

The rationale for including the Bgt_CHN_52_27 was because it was the only one that was fully assembled in one contig. The mitochondrial genome is missing or is fragmented in the others. The unassembled contigs are now supplied separately for isolates (Bgt_CHN_52_27 and Bgt_JPN_CHIKA, see S5 Table). However, the genomes Bgt_CHE_96224 and Bgtl_THUN_12 were already published with the chr-Un. That is why we are keeping them as they are in order to avoid confusion. It has now been clarified in the Main Text (See Lines 288-294).

Reviewer Comment 2.8:
5. Chromosome 11 with characteristic of accessory chromosome
While the authors describe chr11 as having "features of accessory chromosomes," I believe this framing is misleading and detracts from the more biologically meaningful interpretation. Namely, that chr11 may reflect a history of introgression. There is no evidence of presence/absence variation across strains, which is fundamental to defining accessory chromosomes. In fungal genomics, including cases like Zymoseptoria tritici, core chromosomes can undergo significant rearrangements, harbor accessory genes, or result from introgression without being classified as accessory. The emphasis should be placed on the signals of introgression and their evolutionary implications, rather than on drawing parallels with accessory chromosomes, which may not be warranted in this case.
Our response:

While we agree the introgressions may play a role in presence/absence polymorphisms of genes in general, we believe the case of chr11 is different in several aspects. For one, we identified several chromosomal segments larger than 100 kb that are absent in multiple isolates (shown in Fig 4). Additionally, the right arm of chr11 (chr11R) shows overall much higher diversity than the rest of the genome. We performed additional analyses that show higher number of protein variants and gene presence/absence polymorphisms on chr11R. These are presented in a new paragraph (Line 468ff) and a new Fig 5, and Supplementary Figure S18 Fig).

In response to the reviewer's comment, we also toned down the language throughout the manuscript, referring, for example, to “potentially dispensable chromosome segments” (Line 405).

Reviewer Comment 2.9:
6. TE content analysis
The comparison of TE content between reference genomes could be expanded. Are there shared TE families across strains? A clustering approach could identify TE families conserved among strains or specific. This analysis would be particularly insightful for hybrid strains (e.g., the Japanese isolate), potentially revealing "parent"-specific TE inheritance. Additionally, identification of TE families found in the multi-reference context would highlight the added value of this dataset over single-reference approaches as used to detect TIPs.

Our response:

In fact, all TEs in our library were shared between strains, albeit at different abundance levels. We found no evidence for completely new TEs in one or the other isolate, or specific to a group of isolates. We clarify this now in the text (Lines 668ff).

Reviewer Comment 3.5:

Reviewer #3:

For a pan-genome study I found it odd that the authors did not explicitly report or provide a summary or overview of the pangenome size (i.e. what proportion of genes are found in all isolates, core vs accessory genome, and given the discovery of chr11 what proportion of accessory genes are on this chr both within and between forma specialis).

Our response:

In response to comments by reviewer 2, we have constructed a pangenome graph (S13 Fig). From this analysis, we also provide a table with a summary of the sizes, nodes and edges of each chromosome (S6 Table). We also performed additional analysis of gene presence/absence polymorphisms (see also responses to comment 2.4, p.4 and 3.7, p.9)

However, we did not produce a presence/absence matrix for all genes across the 11 PacBio genomes. We manually analyzed numerous loci which show gene presence/absence polymorphisms (see also response to reviewer comments 2.4 and 3.7). This analysis showed us that time-consuming and detailed work is required to sufficiently validate all presence/absence cases, especially in such a highly repetitive genome. We therefore prefer not to use a genome-wide automated approach, as we believe it would not yield reliable enough results.

Reviewer Comment 3.6:

Better integration with existing literature on TEs and fungal pangenomes. Throughout the intro and discussion there are several opportunities to better incorporate existing literature from other pathosystems, for example the long-read Myrtle rust genomes which also have exceptionally high TE content and are also obligate biotrophs. Similarly, the discussion would be strengthened by placing this work into context with other fungal pan-genome studies, for example Fusarium, Pyrenophora tritici-repentis, Verticillium…

Our response:

We have now incorporated more existing literature in the introduction (see Lines 127-128) and in the Discussion (Lines 752-754, and Lines 803-805) as suggested.

Reviewer Comment 3.7:
Copy number variation in pangenome isolates vs. long-read assemblies. Given the authors had some examples of potentially very recent gene duplication events (i.e. E254n in Bgs1459-09640 found in chr-06 and E003 in multiple isolates in chr11, shown in FigS13) I feel it would have been much more informative to look at the location of these genes in the long-read assemblies in more detail, rather than simply report the relatedness of the broader effector family in a phylogenetic tree (Fig5D). The findings of effector gene copy number variation matches reports from other pathosystems, Rhyncosporium secalis, however this study has the added advantage of the long-read assemblies to dig into these copy number variations. For example, how are these extra copies arranged on the chromosome in the long-read assemblies, in-tandem? Do the surrounding genome feature give any hints as to the mechanism driving gene duplication (TE capture, unequal crossing over). Figure 5C and D focuses on very large effector families, but it was unclear to me here within an effector family (E014) which genes had copy number variations.

Our response:

We did additional analyses of gene presence/absence polymorphisms. This includes analysis of chromosomal distribution of gene presence/absence polymorphisms along chromosomes and analysis of number of sequence variants in effector and non-effector genes. The data is described in an additional paragraph (Line 499ff), in a new figure (Fig 5) and a supplementary figure (S18 Fig).

Additionally, we performed detailed manual analysis of selected loci (see also response to comment 2.4, p.4 of reviewer 2 and 3.5, p.8). Indeed, we could show that gene removal was in multiple cases caused by unequal homologous recombination (unequal crossing over) between repeated sequences. We even succeed in all cases to narrow down recombination breakpoints in the sequence that served as template for the unequal recombination. The findings are described in a new paragraph (Lines 617ff), a new figure (Fig 7), and a supplementary figure (S24 Fig). However, we did not identify strict tandem duplications of genes.

Reviewer Comment 3.8:
Line 224-226: Genome size differences were driven largely by "repetitive regions in the centromeres"…where was this data shown-summarised? Figure S3 does not detail the size of the changes, just shows a heat-map of the transposon location and nucleotide (?) similarity in the heatmap legend.
Our response:

Examples were actually shown in Fig 4E through 4H, but were not described sufficiently. Our previous study showed that centromeric regions are defined by absence of genes and high enrichment of *RIX_Fuji* retrotransposons (Müller et al., New Phytologist, 221, 2176-2189). These centromeric regions are clearly visible as repetitive "blocks" which align poorly and differ in size between isolates (Fig 4E through 4H). Centromeres are also visible as regions with an abundance of nodes in the new pangenome graph shown in Fig 5A and supplementary figure S13 Fig (see also response to comments 2.1, p.1 and 2.4, p.4). We describe this now in a new paragraph (Line 457ff), and refer to this section at the place that the reviewer points out (Line 277).

Reviewer Comment 3.9:
Throughout the results and discussion there is reference to a "hybrid" Japanese population with reference to earlier studies. However, in this manuscript this is not given any introduction and therefore makes the reference to this prior work confusing and difficult to interpret.
Our response:

We have now introduced the concept of hybrid populations in Lines 109-113. (see also Reviewer comments 3.21, p.28 and 3.25, p.29).

Reviewer Comment 3.10:
Figure 1C: Authors state that nucleotide diversity was measured in 10 kb windows for eight randomly chosen isolates from populations with at least eight isolates. Should the data identifying the isolates chosen be provided in a supplementary table? (or are these the isolates shown in FigureS2a, if yes this should be stated clearly in the text. Did the authors perform this analysis more than once with different individuals to see if this influenced the results? The ANOVA presented seems to show pair-wise p-values for populations drawn next to each other in the graph, however this does not give a sense of groups (i.e. is the Nuc diversity in Bgdic significantly lower than the USA or ARG?). Would not a

more traditional ab abc bc notation make this analyses more clear?
Our response:

The random isolates were chosen by randomization (in random.org) and are now presented in the first column of the S2 Table and referred to in figure (Fig 1D now) legend, and in the text (Lines 232ff). We have not tried it more than once, since the results with 8 random individuals and all individuals per population show similar results. However, we feel that it might be confusing to group populations purely on the basis of their level of diversity, as it might suggest that, for example, the ARG and *B.g. dicocci* populations are evolutionarily close to one another.

Reviewer Comment 3.11:
Figure 1D: What do the blue, yellow and red colors represent in the background of this plot? Density of data points?
Our response:

Yes, by using the “kde2d” command from the “MASS” package, we estimated the two-Dimensional Kernel Density for the distance. We have now clarified it in the figure. This panel was now moved to supplementary figures (S5 Fig) in response to reviewer comment 2.6, p.6.

Reviewer Comment 3.12:
Figure 2: “except in the highly repetitive centromeric regions” Figure 2 does not show any genomic features that would allow us to assess if synteny breaks are around centromeric sequences.
Our response:

See also our response to comment 3.8. Examples were actually shown in Fig 4E through 4H, but were not described explicitly. Centromeric regions are clearly visible as repetitive “blocks” which align poorly and differ in size between isolates (Fig 4E through 4H). Centromeres are also visible as regions with an abundance of nodes in the new pangenome graph shown in Fig 5A and supplementary figure S13 Fig (see also response to comments 2.1, p.1 and 2.4, p.4). We describe this now in a new paragraph (Line 457ff).

Reviewer Comment 3.13:
Figure 3B and accompanying text 304-315: I did not quite understand the relevance of this section which refers to putative misassemblies. The author

simply state here that they looked re-arrangements close to assembly gaps. However, a much more thorough way to investigate this would be to align your PacBio reads back to your assembly and see if you have reads that span the breakpoints of the translocations. This is easily done with minimap2 or similar software.

Our response:

As suggested by reviewer 1 (see also comment 1.3, p.13), we performed assembly quality control using the CRAQ evaluator, which assessed the quality of the assembly. Now in Lines 269-271, 389-394, and 982-984). This showed high values of contiguity. The data is provided in S4 Table.

Reviewer Comment 3.14:

Figure 4E-H: Given the termini of Chr11 were highly variable both within and between f.s. why were the genes in these regions not investigated in more detail? Did these regions end up in the un-assigned contigs? Or are they truly absent?

Our response:

The termini described in the text are indeed unique to some isolates (e.g. Bgs_1459 or Bgt_CHE_96224, Fig 4E-F). No corresponding segments were found in assembled contigs. Furthermore, the terminal regions of chr11 as the assemblies contain no sequence gaps and CRAQ analysis attested to the robustness of the assembly (see also response to comment 3.13 above). Additionally, the unassembled contigs did not contain any genes.

Reviewer Comment 3.15:

Maybe I missed the download link but only PDF with Supp Figures and Supp dataset 2.xls was available to reviewers. I was unable to review any of the ten supplementary tables, please consider Zenodo.

Our response:

These tables/data have now also been deposited at Zenodo (see also response to Reviewer Comment 3.3, p.3) (see Zenodo link at the last page 30). The table are also submitted along with the manuscript.

Part III – Minor Issues: Editorial and Data Presentation Modifications
Please use this section for editorial suggestions as well as relatively minor modifications of existing data that would enhance clarity.

Reviewer #1: Minor analytical/experimental issues:

Reviewer Comment 1.2:
1. L268ff: mitochondrial genome - is the gene content complete for a fungal mitochondrial genome? Refer to 10.1038/s41598-021-93481-5 and 10.1099/mgen.0.000720 for further information of mitochondrial genomes in powdery mildews.
Our response:

Yes, the gene content appears complete, as it shows perfect collinearity, except for ~5 kb segment that is deleted when compared with the published mitochondrial genome from Bgt_CHE_96224. This seems to be variable region as we find variation in that region also between the two *B. hordei* mitochondria from isolates DH14 and RACE1. This region contains an apparently defective DNA polymerase (type B) gene.

In response to the reviewer's comment, we annotated 3 mitochondrial genomes (the already published mitochondrial genomes for isolates Bgt_CHE_96224 and Bh_DH14) along with the one from this study Bgt_CHN_17_40 with the same pipeline (in Methods, Lines 1022-1028) and obtained very similar gene contents which is now referred to in the text. We also clarify that the mitochondrial genome of Bgt_CHE_96224 was previously published (in Lines 340-344.).

Reviewer Comment 1.3:
2. L304ff: rearrangements. The authors exclusively searched for sequence gaps to identify potential breakpoints. However, much stronger evidence for misassembly errors are to use remapping inconsistencies between the Pacbio reads and the respective assemblies (e.g. with a tool such as CRAQ, which also assesses genome assembly quality and reports read/assembly agreement both locally and globally). In addition, did the authors consider using experimental evidence via chromatin capture-based methods (HiC or PoreC) to correctly arrange all contigs?
Our response:

In the current study we did not have funds for Hi-C, although it would be desirable to have. We are now mentioning it in the discussion as a future

direction (Line 864ff). But as suggested, we now mapped the PacBio reads back to the respective assemblies using CRAQ analysis and did the genome assembly quality assessment. The quality scores are very high which makes us confident in the quality of the assemblies. The data in the quality assessments are provided in S4 Table. We now mention the results of the CRAQ analysis in the section on translocations, which strengthens the discussion of possible mis-assemblies. Nevertheless, we chose to still mention the sequence gap bordering one of the translocations for transparency (Lines 389ff).

Reviewer Comment 1.4:
3. L699ff (material and methods; manual contig scaffolding): If I understand correctly, the authors manually scaffolded the contigs based on the B. graminis f.sp. tritici CHE_96224 reference assembly and introduced arbitrary stretches of N between them (or did they use an automated process such as RagTag? not described). Assembling the scaffolds based on a reference genome risks introducing reference bias in the genome assemblies. How did the authors account or correct for this potential bias?
Our response:

Bias introduced by the reference sequence is indeed a valid concern. To assess the integrity of the assemblies, we ran the CRAQ analysis. As suggested in comment 1.3, p.13. Since all genomes were assembled mostly in Megabase size contigs, we only used the Bgt_CHE_96224 reference genome as a rough guide to arrange contigs. Thus, smaller introgressions and rearrangements in a given isolate did not affect the ordering of the contigs. Together with the results of CRAQ analysis, we are confident that we did not introduce a reference bias.

Reviewer Comment 1.5:
4. L321ff, chr-11 shows characteristics of an accessory chromosome. Did the authors test if any B. hordei DH14 or RACE1 contigs match chr-11, to estimate if this chr-11 is specifically present within the B. graminis lineages or potentially shared within the Blumeria genus?
Our response:

We have identified the homologous chromosome in *B. hordei* DH14 contigs, indicating that a collinear chromosome is conserved between *B.g. tritici* and *B. hordei*. We briefly describe this in the Main Text (Lines 296-298) and show a dot plot alignment in S8 Fig.

Reviewer Comment 1.6:
5. L388: the authors mention that one gene was found only in B. secalis and B.g. triticale. What did this gene code for?
Our response:

This gene is a candidate effector (*Bgs1459-09656* and *BgTH12-07698*). This was only mentioned in S1 Note and S20 Fig. We now also mention it in the main text Line 529 for clarity.

Reviewer Comment 1.7:
6. L494ff, TE distance analysis: this analysis is a little unclear to me, please clarify. In particular, is the analysis limited to TEs adjacent to genes and at what distance cut-off? what are the 21000 TEs on average referring to, those neighboring genes? I think the description would be clearer if it would describe TE distance from the gene point of view, given the TE-enriched nature of the powdery mildew genomes making up the majority of the sequence.
Our response:

The 21,000 copies refer to total annotated full-length TE copies in each genome. For the TE distance analysis, we re-phrased the statements now as suggested from the point of view of genes (Line 662ff).

Reviewer Comment 1.8:
7. It occurs to me that LINEs and SINEs were also among the well-expressed TEs in B. hordei (L510ff). Did the authors use RNA-seq data (public or other) to check if these LINE and SINE elements also exhibit significant expression levels, coinciding with their observed genomic mobility?
Our response:

We have not analyzed transcriptome data with regards to TEs for this study, since we only focused on the genomic resources. Considering the length of the manuscript, we would prefer not to include transcriptomic analysis.

Reviewer Comment 1.9:
8. Methodology: variant calling (L660ff): based on the parameters, it seems there was no filtering by allele frequency (AF vs RF). This may confound the SNV analysis since the variant callers tend to call many SNVs even if the alternative allele frequency is well below 0.8. Did the authors filter by alternative allele

frequency in some way?
Our response:

This is an omission. We have used filtering for Minor Allele Frequency lower than 0.1 as in previous work (Sotiropoulos, et al., 2022, *Nature communications* 13.1: 4315.). We describe it now in the Materials & Methods Lines 927-928.

Reviewer Comment 1.10:
9. L674ff, population genomics analysis: I have tried to understand how this analysis is done, but this section only lists the names of R packages. Please describe the approach used for the population genomics, and/or refer to a public repository with a detailed code collection, so the method can be replicated.
Our response:

We have added more information of the commands used in Materials & Methods to better describe the population genomics approach in Lines 938-963.

Reviewer Comment 1.10:
10. L731ff: TE identification. The authors used manual curation and EDTA to create a TE library. How was genome-wide TE detection done, with RepeatMasker or differently?
Our response:

The genome-wide TE detection was done with RepeatMasker employed by EDTA. This is now mentioned in the methods (Lines 1012-1013).

Reviewer Comment 1.11:
L812-814: the coverage cut-off was 0.1-1.7 (above 1.7 was considered duplicated). Did the authors also consider higher coverage and thus higher level copy number variations? what were the cut-offs for e.g. three, four, five, etc. copies?
Our response:

No, for our analyses, we only distinguished between one or more copies, using the 1.7-fold sequence coverage as cutoff. We now state this in the methods (Line 1108-1111).

Reviewer Comment 1.12:
Minor typographic issues:

Our response:

We give brief responses point-by-point below:

L149: 'about 399 isolates', remove 'about'

- “about” was removed

L166/167: the reads were mapped to the genome or assembly, not the isolate itself. Rephrase.

- corrected as suggested (Line 181)

L212ff: which eight B. graminis isolates exactly were sequenced? Please explicitly mention here (also L694).

- A list of newly sequenced isolates is now provided in the text (Lines 258-259)

L270: remove the word 'us' between 'showed high levels of…'

- ‘us’ was removed

L410: change to 'possible underestimation'

- corrected as suggested (Line 554)

L455: correct 'Transposable Elements insertions' to 'Transposable element insertions'

- corrected as suggested

L506: comma before which missing

- corrected as suggested

L507: 'posing a known bias', which bias? Provide a reference

- the bias comes from shorter reads having less sequence space to align. We state this now (Lines 712-713)

L508-509: something... this statement is very vague and unclear, please rephrase into a precise and clear conclusion.

- the statement was clarified that high TIP numbers in the JPN population can be attributed to the fact that they are hybrids (Lines 714-715)

L517: reference missing for this statement

- We were not sure which statement the reviewer refers to: maybe Line 522 (now 730) “Many factors such as evolutionary pressure or genetic drift could have led to this increase.”, or Line 527 (now 735) “LINEs encode a conserved protein (reverse transcriptase)”. We added references for both statements now (in Line 731 and Line 735).

L563: replace 'from' with 'by'

- Corrected as suggested

L569: 'highest number of variation' do the authors mean 'level'?

- Modified as suggested

L586-587, 'couple less Ma' rephrase into a less colloquial statement

-Rephrased now in Line 808.

L592, 'which is puzzling': colloquial phrase, remove

- this was removed and addressed (see also comments 2.34, p.25, 3.27, p.29 and 3.28, p.30)

L591ff, TE proximity upstream of genes: TEs can also act as promoters for genes, thus increasing expression

- a statement was added to the discussion, mentioning this possibility (Line 819ff)

L597: CITE 4 papers, I believe references are missing here

- this was corrected and expanded (see also comment 3.27, p.29)

Reviewer Comment 2.9:

Reviewer #2: Minor Comments
Line 74: The term “Earlier…” is ambiguous. Please clarify, do you mean earlier in the literature, or earlier in this manuscript?
Our response:

We meant earlier in the literature. We have clarified it now in the text (Lines 77-78).

Reviewer Comment 2.10:
Line 136: The phrase "addition of genes not found by short reads" needs elaboration. Are the authors referring to limitations in short-read based assemblies, or to gene presence/absence undetectable by resequencing? or something else?
Our response:

We have now modified the text to clarify it in Lines 146-148.

Reviewer Comment 2.11:
Line 137: Please explain how using multiple reference genomes improves gene expression analysis compared to a single reference.
Our response:

We have rephrased the statement that differences in expression can be inferred, if a given gene is present in different copy numbers between isolates (e.g. multiple copies of given gene can increase the numbers of transcripts produced) Line 148ff.

Reviewer Comment 2.12:
Line 138: Clarify how "host pangenomes" are relevant to Blumeria genomics.
Our response:

We emphasize now that an important value of the host-pangenomes was that they provided insight into natural diversity, particularly in agriculturally important loci and genes (Lines 153-154).

Reviewer Comment 2.13:
Line 166: Please include citations in Table S1 to support all referenced datasets.
Our response:

We added a column with the references for each individual isolate used (S1 Table, column "Study").

Reviewer Comment 2.14:
Line 195: "statistically less diverse mean" is unclear. Also, was the low diversity in the Australian population due to limited sampling? If PCA shows USA and

Australian populations overlap, are they truly isolated?
Our response:

We have now clarified the “statistically less diverse mean” section in Lines 224-226, 234-237. Australian powdery mildew does not look isolated (more isolates are needed), but we are grouping it separately for geographic grouping purposes in some cases. We are also explaining further what is happening to the Australian powdery mildew isolates in Lines 241-242.

Reviewer Comment 2.15:
Lines 201–202: The phrase “as hypothesised before” is more appropriate for the discussion. The results section reads as a re-confirmation of previous findings, raising concerns about novelty.
Our response:

We have now placed this statement in the Discussion (Lines 768-772).

Reviewer Comment 2.16:
Lines 204–209: Clarify how geographic groups were defined for the Mantel test. Explicitly state conclusions about spatial vs genetic distance correlations. Could trade explain the observed patterns?
Our response:

We have now defined the geographic groups in S3 Table. We are also now explicitly stating the conclusions about the correlations. Trade could be one of the reasons that could explain the observed patterns, now mentioned in Lines 247-248.

Reviewer Comment 2.17:
Figure 1:
In 1A, the triangle symbol for B. graminis dicocci is indistinct.
In 1B, are both dicocci and tritici isolates shown? Do formae speciales cluster separately? What does this imply about host specificity?
In 1C, clarify whether the p-values shown represent all or only significant pairwise comparisons. The figure legend is unclear. What does “based on 8 randomly chosen isolates” mean?
Our response:

For 1A, we understand, we have now removed these symbols as they are not very informative.

For 1B, only *B.g. tritici* isolates are used and shown. The other formae speciales are very different, and we would lose the resolution of the PCA from the majority of isolates. We now state this in the Figure legend. We also added information to the Figure legend and the text (Lines 232-233) how isolates were randomly chosen.

For 1C (now 1D), we have clarified the p-values in Lines 224-226.

Reviewer Comment 2.18:
Table S1: Please include references for each dataset. Were any new Illumina reads generated for this study? This was not clearly stated.
Our response:

We added a column with the references for each individual isolate used (S1 Table, column "Study"). No, new Illumina data were not generated, except from the ones for the pangenome isolates (for quality purposes). Now clarified in Lines 892-893.

Reviewer Comment 2.19:
Figure S6: Define "grouped" vs. "ungrouped" effectors. Do you mean effector multigene families?
Our response:

Yes, we mean known effector families. This is now clarified in the legend in S9 Fig.

Reviewer Comment 2.20:
Figure S7A: Consider ordering the x-axis by chromosome number for readability.
Our response:

We have now re-ordered the x-axis by chromosomes in S10 Fig.

Reviewer Comment 2.21:
Line 220: Using the term "pangenome" is misleading if a pangenome graph was not created. Please clarify.
Our response:

A pangenome graph has now been created. We have included it in Fig 5A and S13 Fig. Also, in line with Reviewer Comments 2.1, p.1, 2.3, p.2. Now mentioned in Lines 306-312.

Reviewer Comment 2.22:
Line 226: Are centromeric repeats the sole drivers of genome size variation? How was this determined?
Our response:

Centromeric repeats are not the sole driver of genome size variation, but do indeed explain most of it. In fact, we show examples Fig 4E through 4H. These centromeric regions are clearly visible as repetitive “blocks” which align poorly and differ in size between isolates (see also response to comments 2.1, p.1, 2.4, p.4 and 3.12, p.11). We dedicated now a short paragraph to centromeres (Line 457ff).

Reviewer Comment 2.23:
Line 239: Was chr_Un generated by merging unassembled contigs? If so, this may be misleading. Unplaced contigs should remain as such rather than being artificially grouped.
Our response:

Any contig that was merged into a chr-Un has now been split again into unassembled contigs in S5 Table. However, for the chr-Un of isolates Bgt_CHE_96224 and Bgtl_THUN_12, we did not change them, because they have been published like this.

Reviewer Comment 2.24:
Line 240: Why is part of the mitochondrial genome found in chr_Un? Normally, mitochondrial genomes are assembled and analyzed separately.
Our response:

This refers to a previous publication where the mitochondrion of Bgt_CHE_96224 was initially placed on chr-Un and analyzed in a later publication. We now clarify this and added the appropriate reference (Lines 335ff).

Reviewer Comment 2.25:
Line 245: The phrase “which shows the potential indication of recombination” is

vague. What precisely indicates recombination here?
Our response:

A hybrid node indicates recombination. Now rephrased in Lines 301-304.

Reviewer Comment 2.26:
Line 246: What observation is being referred to regarding Bgt_JPN? Please clarify.
Our response:

The observation of both Bgt_JPN_CHIKA and the Bgtl_THUN-12 having hybrid nodes. This was re-written for clarification in Lines 301-304.

Reviewer Comment 2.27:
Lines 273–279: Are these loci consistently located at the same positions across all assembled genomes? While I would expect them to be conserved, it would be interesting to know whether any structural rearrangements have occurred in the regions surrounding the mating-type loci. Given the potential implications for recombination and reproductive isolation, this could be an informative aspect to address, particularly in the context of genome structure variation.
Our response:

The mating type locus is on chromosome 1. Sequences flanking the mating type loci are highly collinear between isolates of the same mating type, with the exception of occasional TE polymorphisms. In contrast, a region of ~200 kb surrounding the mating type genes do not align between isolates of different mating types. We have added this information (Line 351ff).

Reviewer Comment 2.28:
Lines 306–307: Clarify which rearrangement is being referenced.
Our response:

We have clarified it now in Lines 389-394.

Reviewer Comment 2.29:
Line 400: It is unclear which reference genome was used to assess copy number variations (CNVs). Did the authors use a single reference genome for all populations, or were population-specific references employed? If only one reference was used, it raises the question of how leveraging multiple high-quality

genomes in a pangenome framework adds value to the CNV analysis. A key advantage of a pangenome approach is the ability to reduce reference bias and better capture structural variation across diverse lineages—this benefit would be diminished if CNVs are mapped solely to a single genome.

Our response:

We only used a the published Bgt_CHE_96224 reference genome for all populations to assess the copy number variations. We state this now in the text (Line 540).

But the reviewer is correct in that this is a limitation of the analysis, as it only can cover sequences present in the Bgt_CHE_96224 reference. However, we found a genome-wide all-vs-all comparison would be too complex in such a repetitive genome. We instead resorted to the detailed analysis of a small number of loci that show presence/absence polymorphisms (see response to comment 2.4, p.4 and 3.7, p.9).

Reviewer Comment 2.30:

Line 448: Is AvrPm2 present in all isolates? Is there any host specialization linked to its presence or absence?

Our response:

As studied before (Praz et al. 2017, and Jaegle et al. 2025), *AvrPm2* absence is associated with virulence. We did a blast search of this gene and two other genes as queries in the same effector family (as done before for other isolates) using the pangenome assemblies as databases. *AvrPm2* is present in all, but two isolates of the *B. graminis* pangenome (Bgt_CHN_52_27 and Bgtl_THUN_12). This is now included in Lines 596-598 and in S10 Table.

Reviewer Comment 2.31:

Line 520: Was this TE family with high number of TIPs in the population, also depicts a high TE copy number in the TE annotation of the reference assembly of Bg dicocci?

Our response:

Yes, it does have a higher TE copy number in the TE annotation of the reference assembly of the *B.g. dicocci* compared to the other genomes. This was now added in S13 Table and in the main text in Lines 727-729.

Reviewer Comment 2.32:
Line 542: The statement linking accessory chromosomes to virulence in Z. tritici is misleading and should be revised. In this species, accessory chromosomes are not directly associated with virulence. In fact, studies have shown that the loss of certain accessory chromosomes can even enhance virulence on specific hosts (e.g., Habig et al., 2017).
Our response:

We have now added this reference and revised the statement. Now in Lines 749,751.

Reviewer Comment 2.33:
Line 567: The wide variation in chr11 size and introgression suggests large-scale structural rearrangement, contrary to this sentence stating of no major rearrangements between Bg genomes.
Our response:

We have now rephrased the statement in Lines 781-786.

Reviewer Comment 2.34:
Lines 591–592: The observed proximity of TEs to effector genes is a well-documented feature in many fungal plant pathogens. However, the authors should also acknowledge that overlap between TE and effector annotations can sometimes result from annotation artifacts, particularly in repeat-rich genomes. It would be prudent to mention that such cases may require manual curation to distinguish true gene–TE associations from misannotations.
Our response:

We now mention the possibility of annotation artifacts in the discussion (Lines 815-817). However, we also emphasize that the reference genome Bgt_CHE_96224 had over 1,300 effector genes manually curated in a previous study for its effectors (Mueller et al. 2019). See also responses to Reviewer comments 3.27, p.29 and 1.12, p.16.

Reviewer Comment 2.35:
Line 597: Please replace “(CITE 4 paper)” with the actual references.
Our response:

We have now replaced the error and included references in Line 825.

Reviewer Comment 2.36:
Line 612: The authors may wish to include a brief discussion contrasting pangenome graphs with the multi-reference genome approach used in this study. While the availability of multiple high-quality assemblies is undoubtedly valuable, a pangenome graph offers a structured framework for representing gene content and structural variation across strains, enabling more systematic comparative analyses. Discussing the trade-offs between these approaches (such as resolution, computational complexity, and biological interpretability) would help clarify the scope and limitations of the current work, and situate it more clearly within the broader context of pangenomic research.

Our response:

We agree with the reviewer that a pangenome graph offers distinct perspectives and insights. We have produced a pangenome graph and present it in results and figures (see responses to comments above). However, considering the length of the manuscript, we would prefer to not engage in more in-depth discussion and exploration of the merits of pan-genome graphs in comparison to other approaches.

Reviewer Comment 3.16:

Reviewer #3: The authors report a high level of synteny between the long-read genome assemblies. In Fig3 several large-scale rearrangements are reported that contain dozens of genes. Did the authors investigate what genes these were and/or the flanking regions to better understand 1) what may have driven the rearrangement 2) do these genes appear to have important functions that would require co-regulation (secondary metabolites).
Our response:

We did not perform detailed analyses of these loci. Considering the length of the manuscript, we would prefer to leave that for a dedicated follow-up study. We state this now in the discussion (Line 791ff).

Reviewer Comment 3.17:
Lines 119-120 and 127-128:There are some gaps in the introduction in terms of recent publications from many rust fungi (also an obligate biotroph) that have also been shown to have very high TE content

(https://doi.org/10.1093/g3journal/jkaa015).
Our response:

It is now mentioned in the introduction Lines 127-128 and Line 136.

Reviewer Comment 3.18:
Line 207: “broader region of origin” can you be more explicit in this sentence about which populations you included here (“Middle Asia” in Figure 1D)
Our response:

We now include the names of the isolates in S3 Table. Also, the names of the populations used are mentioned in the main text (Lines 246-248 and Line 250-251).

Reviewer Comment 3.19:
Line 216 and Fig1A and B: It would be helpful to know where isolate THUN-12 is from? Why are ISR_7 and CHE-96624 highlighted in the map but not this other isolate? Also there are nine names highlighted in Figure 1A, only eight names highlighted in Figure1B but eleven isolates sequenced with long reads (two B.g. dicocci names not shown?). Please revise to be consistent.
Our response:

Isolate THUN-12 is from Poland. It is mentioned in S1 Table.

For Figure 1A, we only highlighted the *B.g. tritici* and *B.g. dicocci* isolates in the map. *B.g. triticale* THUN-12 is a hybrid of rye and wheat powdery mildew, and B.g. secalis Bgs_1459 is a different forma specialis for which we have less information on their collection. We have now added them to the figure.

For Figure 1B, we only included *B.g. tritici* isolates as the other three isolates Bgtl_THUN-12, Bgd_ISR211, and *B.g. secalis* Bgs_1459, are more distant and we would lose the resolution of the majority of the isolates in the PCA. We now state this in the figure legend.

Reviewer Comment 3.20:
Line 217: Please name country of origin for B. hordei isolate.
Our response:

We now state that the *B. hordei* isolate originates from England (Line 264) and provided the appropriate reference (Hacquard, Stéphane, et al. *Proceedings of the National Academy of Sciences* 110.24 (2013): E2219-E2228.).

Reviewer Comment 3.21:
Line 246: why was Bgt_JPN_CHIKA expected to be an outgroup with the other isolates in Figure S7? It appears to group within the main cluster of Bgt isolates, so this sentence does not make sense. Also in this figure what do the different colors mean? Population of isolation?
Our response:

Isolate Bgt_JPN_CHIKA is expected to be a hybrid (not an outgroup) between two *B.g. tritici* parent (coming from the USA and the CHN powdery mildew populations respectively), while Bgtl_THUN_12 is also a hybrid, but with parents coming from two different formae speciales (one from *B.g. tritici* and one from *B.g. secalis*). Now explained and clarified in Lines 109-113 and Lines 301-304. In line with Reviewer comment 3.9, p.10 and 3.25, p.29.

Reviewer Comment 3.22:
Lines 255-256: The referral to B hordei DH14 in the figure legend for Figure2 appears slightly out of context. Is this the most appropriate place for this? There is not accompanying text in the main results for this S8 figure.
Our response:

We agree with the reviewer that the sentence is without context and removed it from the figure legend. Nevertheless, as the closest related species to *B. graminis,* we wanted to include this comparison for completeness purposes. We now mention it briefly in the main text (Line 296ff) and now in S11 Fig.

Reviewer Comment 3.23:
Line 259-262: run on sentence, please revise.
Our response:

We have now revised the sentence (Line 324-328).

Reviewer Comment 3.24:
Line 279: Mating type analysis, can you please detail which isolates belong to

which mating type
Our response:

The details are presented in S9 Table. Supplementary Tables file was also uploaded to Zenodo (see Zenodo link at the last page 30).

Reviewer Comment 3.25:
Line 284-289: This section of the results refers to some previous published (?, ref7?) knowledge of the Japanese population being a hybrid of a US and CHN isolate. It would be helpful if this could be more thoroughly introduced or placed into context before discussing these genome translocations.
Our response:

This is now presented in the beginning in the Introduction in Lines 109-113. (see also response to comments 3.9, p.10 and 3.21, p.28).

Reviewer Comment 3.26:
Line 552-553: "our work has supported" this sentence does not give details as to which data-results from this work supports the use of the new naming nomenclature, it would be good to be more explicit here and refer to supporting data (i.e. whole genome synteny and accessory chr11)
Our response:

We have now rephrased the statement in accordance with the comment (Lines 762-763).

Reviewer Comment 3.27:
Line 593-597: I was surprised that the authors refer to the close association of effector genes to TEs as "puzzling", given this has been reported for many fungal species. Please see https://www.sciencedirect.com/science/article/abs/pii/S0168952521002298 for a comprehensive review. Also this section concludes with (CITE 4 papers).
Our response:

This "puzzling" statement as been removed. Instead, we include now the suggested citation (Line 815). We also expanded the discussion on the association of effectors with TEs substantially, also in response to comments 1.12, p.18, 2.34, p.25 and 3.28, p.30 (Lines 813-822).

Reviewer Comment 3.28:
Pangenome analyses of many pathogenic fungi has also been performed: see Linked to this is a suggestion that the Discussion could certainly be improved and made more accessible to the wider evolutionary biology community by discussing the TE content of these fungi in the context of their obligate biotrophic lifestyle.

Our response:

The reviewer raises the important point of whether the absence/loss of RIP is actually an essential requirement in the evolution of obligate biotrophy, if a fungus has RIP, it would hamper processes of gene duplication and deletion as well as the evolution of novel alleles through unequal recombination. We discuss this point now (Lines 848ff).

Zenodo link:

https://zenodo.org/records/14020735
