# Supplementary material for: Combining pangenomics and population genetics finds chromosomal re-arrangements, diversified chromosome segments, copy number variations and transposon polymorphisms in wheat and rye powdery mildew
Source: PLoS Pathog. 2026 Apr 20;22(4):e1013196. doi: 10.1371/journal.ppat.1013196 (PMC13124064; doi:10.1371/journal.ppat.1013196)
Supplement: Attachment — Submitted filename: Response to Reviewers Rev2.docx [file ppat.1013196.s005.docx]

**Reviewers' Comments:**

Reviewer's Responses to Questions

**Part I - Summary**
Please use this section to discuss strengths/weaknesses of study, novelty/significance, general execution and scholarship.

Reviewer #1: The manuscript has been much improved by the revisions. All my concerns have been addressed. I particularly appreciate the inclusion of pangenome graphs and analyses, providing more depth and better formalizing the findings in wheat powdery mildew.

Reviewer #2: The authors have thoroughly addressed all of my comments. The revised manuscript reflects substantial improvements in clarity, methodological detail, and interpretation. I appreciate the authors’ efforts in providing comprehensive responses and implementing the requested changes, which have strengthened the overall quality of the work.

Reviewer #3: This is a revised manuscript which I have previously reviewed. While the authors have made a significant number of changes and improved the manuscript, I feel they have chosen to not incorporate some major suggestions made independently by multiple reviewers. I disagree with some of their rational for not incorporating some changes and try to provide some detail on this below.

**Our response:**

We would like to thank the reviewers for appreciating our revision. We have now revised the manuscript following the comments and suggestions by the reviewers. Below, we provide point-by-point answers to the comments.

**Part II – Major Issues: Key Experiments Required for Acceptance**
Please use this section to detail the key new experiments or modifications of existing experiments that should be absolutely required to validate study conclusions.

Generally, there should be no more than 3 such required experiments or major modifications for a "Major Revision" recommendation. If more than 3 experiments are necessary to validate the study conclusions, then you are encouraged to recommend "Reject".

Reviewer #1: (No Response)

Reviewer #2: (No Response)

**Reviewer comment 3.1:**

Reviewer #3: I think the largest omission is still that the pangenome in terms of the proportion of core, softcore and fully accessory genes are still not reported, which the authors respond by saying the genome is too repetitive to achieve in an automated way with accuracy. While I agree with the authors that it is difficult to manually validate every gene presence absence polymorphism, I would also argue that having 100% accuracy in calling a gene present or absent in a pangenome is not expected (as the addition of new isolates could always highlight new “accessory” genes). I can only re-iterate my opinion that a pangenome study would attempt to quantify what proportion of genes are present in all isolates, a large fraction and genes that are singletons. I recognise that this comes with some degree of uncertainty. I believe the authors do have this data to hand (i.e. the genome annotations for each isolate), so I do not feel this is a major analyses to add in and would greatly benefit the study. The authors do present a pan-genome graph (as requested by another reviewer), showing areas of the genome that are more variable than others, primarily showing lots of heterogeneity around repetitive centromeric sequences. I guess I fail to see how these pangenome graphs really help drive our understanding of this pathogen, without some sort of more detailed analysis of the gene content within these loops.
**Our response:**

In response to the reviewer’s comment, we performed a comparative analysis to estimate the sizes of core and accessory genomes. After using the Liftoff software (Shumate and Salzberg, Bioinformatics. 2021, 37:1639-1643) to detect genes that may have been missing in some of the annotated isolates, we cross-matched all coding sequences (CDS) of the sequenced isolates. We used bi-directional blastn searches to identify genes that are conserved across all isolates. Here, we analysed candidate effector and non-effector genes separately, because effectors represent the adaptive part of the powdery mildew genome. We derived sample curves to extrapolate the sizes of the core candidate effector and non-effector genes. Additionally, we did this extrapolation twice, once including and once excluding the *B.g. secalis* isolate 1459. Interestingly, the size of the core non-effector genome differed by only ~2% while the size of the core effector genome differed by ~15% when *B.g. secalis* isolate 1459 was in- or excluded. For this analysis, we collaborated with Dr. Edith Schlagenhauf (University of Zurich), whom we would like to include as additional author for this study. The results of the analysis are described in the main text (Lines 589-620) under a new sub header and in Figure 6C-E. The previous Fig 6C and the corresponding text were moved down since they topically fit well after the new material. The previous panel Fig 6D was moved to the supplementary material (new Fig S25).

The procedure is described in a new section in the methods (Lines 1164-1196). Additionally, we provide all R scripts and original Perl scripts on our GitHub repository (github.com/Wicker-Lab/Blumeria_pangenome). There, we also provide detailed descriptions on how the custom Perl scripts work and how to use them.

**Reviewer comment 3.2:**
Throughout the manuscript and especially towards the end of the results the grouping of candidate effectors on Chr11 is discussed. I found it difficult to understand if these are “candidate effectors” predicted based on some software or expression timing or if they were validated effectors. I noticed there was some inconsistency throughout the manuscript as to how these genes are referred to, in some cases “candidate” is used and in other cases not. The authors refer to a previous study that validated or classified these genes but I found this introduction quite late in the results. I wonder if this could be introduced earlier to clarify what this gene set is and whether or not these are still “candidate” or not. I also felt that these genes could be highlighted earlier in the manuscript which might help build towards the final results section looking more specifically at the presence absence of these genes. For example highlighting the (candidate) effectors locations in Fig2 (see below).

**Our response:**

The language concerning candidate effectors was indeed inconsistent. Past studies have identified and functionally validated a number of candidate effector genes as avirulence genes (*Avrs*) or suppressors of avirulence genes in *B.g. tritici.* However, *Blumeria* genomes contain 800-1,000 candidate effector genes which were identified based on protein homology to confirmed Avrs and characteristic protein domains such as a signal peptide (Müller, Praz et al. New Phytologist*.* 2019, 221(4): 2176-2189). We now introduce validation of Avr proteins earlier in the text. We also define “candidate effectors” accordingly early in the introduction in Lines 78-85 and state clearly that we will refer to them as “effectors” thereafter except in cases where we refer to, for example, confirmed Avr genes/proteins. We now also highlight the effectors in Fig 2 as well (see also Reviewer’s comment 3.3).

**Reviewer comment 3.3:**
Reviewer Comment 3.12: Figure 2 remains unchanged
I do not feel that this comment has been addressed as Figure 2 remains unaltered. I think it is a relatively simple addition to add the centromere’s to Figure 2 that would enhance the manuscript and enable the text as written to match the Figure. While Figure 4 does show a lack of synteny around the centromere as indicated, why refer to this before the data is actually presented? Another enhancement of Figure2 I think would be to color the gene lines based on genes vs. candidate effectors. This would enable a global comparison of effector gene clustering on Chr11 which becomes much more important later in the manuscript.
**Our response:**

We have revised the synteny presentation in Figure 2, now including the positions of centromeres and effector genes as suggested (see also Reviewer’s comment 3.2). We would prefer not to display all the genes, because indicating all the ~9,000 genes in the figure would result in a contiguous bar across chromosomes, except in centromeres (see an example below).

We found it appropriate to refer to the later data at this point, as they provide the explanation for the size differences in chromosomes (highly polymorphic TE insertions in centromeres). Since it would disrupt the manuscript flow to present the details on centromeres here, we just want to point out to the reader that the detailed explanation will come later in the manuscript.

The Figure above shows an example for part of the synteny plot where positions of all genes are indicated with light blue vertical bars. Note that individual bars cannot be distinguished due to the high number of genes.

**Reviewer comment 3.4:**
Code used for R analyses and whole genome alignments is not provided. I think it is reasonable to ask for all code used to perform the population genetics and genome alignments to be provided as separate scripts in either the Zenodo repository or on GitHub. This is standard practice now within the field.
**Our response:**

We now deposited all R and original Perl scripts in our GitHub repository (github.com/Wicker-Lab/Blumeria_pangenome). We provide all R scripts that were used to produce the graphs in the main and supplementary figures, as well as the underlying data used for the plots. For Perl scripts, we provide detailed explanations of the process how they were used (see also comments 3.5 and 3.6 below).

Large datasets such as variant call files (vcfs) were deposited at Zenodo (https://zenodo.org/records/18946991).

This is now also stated in the methods in the dedicated section “Code availability” (Lines 1238ff).

**Reviewer comment 3.5:**
Linked to this comment the authors Methods lack details into how the synteny plots were produced, they refer to the plotting tool itself (NGenomeSym) but how the alignments between the genomes were generated remains slightly unclear. NGenomeSyn provides a few suggested methods for performing whole genome alignment including Mummer and Minimap2, but the authors do not state clearly what method was used. The code used is not provided so it is not possible for me to ascertain this information from the methods.
**Our response:**

We used Mummer with NgenomeSyn. The code is the standard suggested by NgenomeSyn. We now clarified and detailed this in the Methods in Lines 1080-1085.

**Reviewer comment 3.6:**
For the analysis of the sequence conservation accross isolates the authors provide a vague description of how they used blastn of 1kb segments to map synteny across the isolates. Again the code here is not provided and there are some important ommisions. For example blastn is quite sensitive so aside from looking at the “top blastn hit within 5% of its position in the reference chromosome”, were other thresholds used, for example query coverage and percent id? I would request that this custom blast script be provided for reproducibility.
**Our response:**

The blastn hits were indeed examined but this was not stated explicitly. Since the genomes examined are overall very similar, we did not have any top blastn hits shorter than 300 bp. Even between formae speciales (e.g. between Bgt_CHE_96224 and Bgs_1459), ~94% of top blast hits were longer than 500 bp and ~79% were longer than 900 bp. We therefore did not use any filtering for hit size. We only filtered for orientation, and as previously described, for this that are positioned within 5% of their position in the reference chromosome. We have added this information now to the methods.

Additionally, we now provide a detailed description of the Perl scripts used to construct the plots in Figure 4B-4H on our GitHub page (github.com/Wicker-Lab/Blumeria_pangenome). We describe the logic and workflow of the Perl scripts in the accompanying README file and the Perl scripts themselves contain numerous comments. We also provide the underlying source data (blastn hit tables). This allows reproduction of the graphs.

**Reviewer comment 3.7:**
Reviewer comment 2.7: Chr-Un
I agree with the other reviewers comment that artificially fusing contigs into a chimeric chromosome Un should not be done (i.e. has no biological meaning) and am surprised that the previous publications were allowed to do this (a small gap in peer review it seems). I think it would be worth pointing this out as politely as possible that this is problematic. Could the authors even use this manuscript to make the correction? Chr-Un still appears in many of the supplementary figures, which I would suggest removing throughout the manuscript.
**Our response:**

We agree that the use of a chr-Un is problematic and not biologically informative. We have now removed chr-Un from the figures (Supplementary Figures 10 & 11) and text references to it, as suggested by the reviewer. We only refer to it now in the Supplementary Table 5 for completeness.

Although it is not permitted nowadays anymore, it was at the time of publication of the original manuscript on the Bgt_CHE_96224 reference genome. We wish it were not so. However, the process of providing a corrected version might take months and require a complete resubmission of a new version of the genome. We do not see enough benefit, especially, since we did not include chr-Un in any analysis. All newer sequenced genomes do not have chr-Un (see Supplementary Table 5).

For completeness, the chr-Un of Bgt_CHE_96224 is still included in S5 table in line with Reviewer Comment 3.8, but we explicitly state on the Table that chr-Un is a “Collection of unordered sequence contigs that are separated by a span of 200 Ns”.

**Reviewer comment 3.8:**
The methods lines 948-950 in the new manuscript still contain a description of combining unplaced contigs, which I think should be removed. “Contigs with no blast hit were gathered into a chromosome Unknown” same again lines 987-898 “pieces of the mitochondrial“genome were scattered in Bgt_chr-Un which we eventually did not use, while for the latter, the mitochondrial genome was in one contig”
**Our response:**

The first sentence referred to was indeed wrongly placed and has been removed as we are not using chr-Un and the text revised (Lines 1019-1020).

We also removed the sentences referring to mitochondrial fragments in chr-Un of Bgt_CHE_96224 (Lines 292ff and 1060-1062), since we did not analyze them further. In line with Reviewer’s comment 3.7.

**Reviewer comment 3.9:**
Revised Discussion: While I appreciate that the authors have taken the reviewer comments on-board. I do not find some of these comments/ideas well integrated into the discussion and that the discussion still largely lacks a logical structure that is easy to follow. For example, in the first paragraph, they have added three examples of pan-genome studies and listed their general findings and simply follow this with a statement that pan-genomes from biotrophs are lacking. This is followed by a paragraph on host-specificity, followed by another paragraph that repeats some of the discussion on accessory chromosomes.

**Our response:**

We agree with the reviewer that the beginning of the discussion was not particularly well structured. We have now re-organized especially the beginning part of the discussion to increase the flow of the text. Namely, we moved the part with the three examples of pan-genome studies down to the section where accessory sequences are discussed and where it fits better. We also improved some transitional sentences which help improve the flow (Lines 781-822).

**Part III – Minor Issues: Editorial and Data Presentation Modifications**
Please use this section for editorial suggestions as well as relatively minor modifications of existing data that would enhance clarity.

Reviewer #1: (No Response)

Reviewer #2: (No Response)

Reviewer #3: New Comments:

**Reviewer comment 3.10:**

Line 385-386: “In B.g. tritici isolates, chromosomes 1 through 10 show generally high sequence conservation”… Generally “high” sequence conservation is very generic, can this be more precisely presented with an average sequence identity using average ANI values presented in Table S7?

**Our response:**

In addition to the genome-wide ANI values, we have now performed the ANI analyses independently for each chromosome and created heatmaps showing the ANI values between the pairs now in S16 Fig. Additionally, we also state the median values in the text instead of the generic statement (Lines 395ff).

**Reviewer comment 3.11:**
Line 722-72: Various used twice in same sentence, consider revising.
**Our response:**

It has been revised and changed now in Lines 810ff.

**Reviewer comment 3.12:**

Methods:
FigS6: RII_Fuji is labelled RIX_Fuji in the main text (was corrected during revision) but remains RII in Supp materials.

**Our response:**

It should be RII since we know it is a LINE “I” superfamily transposable element (RIX refers to a LINE element where the superfamily is unknown). It has been now revised in the Main text in Lines 280 and 452-455.

**Reviewer comment 3.13:**
FigS8: Why does Bgt_CHE_96224 have three versions for the BUSCO cds scores?
**Our response:**

These were from different versions of annotation as the Bgt_CHE_96224 reference genome has undergone multiple versions of manual curation over time. We have removed older versions and only kept the annotation we have used in S8 Fig.

**Reviewer comment 3.14:**

FigS9A: Green “ungrouped” effectors are not visible in this graph. Assuming numbers below indicate the number of genes? Perhaps an inset or a clearer legend could show these.

**Our response:**

Now they are indicated as suggested in FigS9A.

**Reviewer comment 3.15:**
FigS11: There are still 12 Chromosomes in this figure. Assuming 12th is Chr-Un which should be removed.

**Our response:**

Yes, this was Chr-Un. It has now been removed from S11 Fig.

**Reviewer comment 3.16:**
FigS14A: Why do you have chloroplast (plant organelle) related genes in this figure legend?

**Our response:**

The legend included all genes related to any organelle directly from the organelle annotation software (OrganellarGenomeDRAW). Genes not shown in the figure have now been removed from the legend in FigS14A.
